# Prospective Proteomic Study Identifies Potential Circulating Protein Biomarkers for Colorectal Cancer Risk

**DOI:** 10.3390/cancers14133261

**Published:** 2022-07-03

**Authors:** Xiaohui Sun, Xiao-Ou Shu, Qing Lan, Monika Laszkowska, Qiuyin Cai, Nathaniel Rothman, Wanqing Wen, Wei Zheng, Xiang Shu

**Affiliations:** 1 Department of Epidemiology & Biostatistics, Memorial Sloan Kettering Cancer Center, New York, NY 10017, USA; sunx3@mskcc.org (X.S.); laszkowm@mskcc.org (M.L.); 2 Department of Epidemiology, Zhejiang Chinese Medical University, Hangzhou 310053, China; 3 Division of Epidemiology, Department of Medicine, Vanderbilt-Ingram Cancer Center, Vanderbilt University Medical Center, Nashville, TN 37232, USA; xiao-ou.shu@vumc.org (X.-O.S.); qiuyin.cai@vumc.org (Q.C.); wanqing.wen@vumc.org (W.W.); wei.zheng@vanderbilt.edu (W.Z.); 4 Division of Cancer Epidemiology and Genetics, Occupational and Environmental Epidemiology Branch, National Cancer Institute, Rockville, MD 20850, USA; qingl@mail.nih.gov (Q.L.); rothmann@mail.nih.gov (N.R.)

**Keywords:** colorectal cancer risk, circulating proteomics, biomarkers, nested case-control study, Shanghai Women’s Health Study

## Abstract

**Simple Summary:**

Studies on circulating protein for colorectal cancer risk in a prospective study design is lacking. The aim of the present study was to scan and identify the protein markers by using proteomics technologies in a two-stage case-control study nested within the Shanghai Women’s Health Study (SWHS), a population-based prospective cohort study. In the discovery set, we found 27 circulating proteins with a nominally significant association. Six of them, including CD79B, DDR1, EFNA4, FLRT2, LTA4H, and NCR1, were validated in the validation phase of the study. This study is the first to evaluate over 1000 circulating proteins in prediagnostic blood samples for their associations with CRC risk in East Asians.

**Abstract:**

Background: Proteomics-based technologies are emerging tools used for cancer biomarker discovery. Limited prospective studies have been conducted to evaluate the role of circulating proteins in colorectal cancer (CRC) development. Methods: A two-stage case-control proteomics study nested in the Shanghai Women’s Health Study was conducted. A total of 1104 circulating proteins were measured in the discovery phase, consisting of 100 incident CRC cases and 100 individually matched controls. An additional 60 case-control pairs were selected for validation. Protein profiling at both stages was completed using the Olink platforms. Conditional logistic regression was used to evaluate the associations between circulating proteins and CRC risk. The elastic net method was employed to develop a protein score for CRC risk. Results: In the discovery set, 27 proteins showed a nominally significant association with CRC risk, among which 22 were positively and 5 were inversely associated. Six of the 27 protein markers were significantly associated with CRC risk in the validation set. In the analysis of pooled discovery and validation sets, odds ratios (ORs) per standard deviation (SD) increase in levels of these proteins were 1.54 (95% confidence interval (CI): 1.15–2.06) for CD79B; 1.71 (95% CI: 1.24–2.34) for DDR1; 2.04 (95% CI: 1.39–3.01) for EFNA4; 1.54 (95% CI: 1.16–2.02) for FLRT2; 2.09 (95% CI: 1.47–2.98) for LTA4H and 1.88 (95% CI: 1.35–2.62) for NCR1. Sensitivity analyses showed consistent associations for all proteins with the exclusion of cases diagnosed within the first two years after the cohort enrollment, except for CD79B. Furthermore, a five-protein score was developed based on the six proteins identified and showed significant associations with CRC risk in both discovery and validation sets (Discovery: OR_1-SD_ = 2.46, 95% CI: 1.53–3.95; validation: OR_1-SD_ = 4.16, 95% CI: 1.92–8.99). Conclusions: A panel of five protein markers was identified as potential biomarkers for CRC risk. Our findings provide novel insights into the etiology of CRC and may facilitate the risk assessment of the malignancy.

## 1. Introduction

Colorectal cancer (CRC) is the third most diagnosed and second common cause of cancer death worldwide, with approximately 1.9 million new cases and 0.9 million deaths globally in 2020 [1,2]. It is estimated that the global CRC burden will increase by 60% by 2030, largely attributable to the rapid rise of its incidence in low-income and middle-income countries [3]. Although genetic predisposition factors and modifiable risk factors such as obesity, physical inactivity, smoking, excess alcohol consumption, high intake of red and processed meat, have been identified for CRC [4,5], the etiology of CRC is not fully understood.

Proteins are critical players for nearly all essential biological processes in the human body [6]. Deregulated proteins, such as cytokines, chemokines, and matrix-degrading enzymes, have been shown to play an important role in the tumor microenvironment [7,8,9]. To facilitate the current understanding of CRC etiology, previous studies have assessed the potential of circulating proteins as risk factors or biomarkers for CRC. For instance, multiple population-based studies reported significant associations of circulating proteins in response to systemic inflammation with CRC risk [10,11,12,13]. Studies also linked proteins involved in the insulin and insulin-like growth factor (IGF) signaling pathways to CRC development [14], supporting the mechanistic roles of obesity and insulin resistance in colorectal tumorigenesis. However, the candidate approach employed in those studies is restricted to proteins for which extensive prior knowledge of the mechanistic pathways is required, making the application to majority of proteins in the human body challenging.

Given the minimally invasive nature of the approach used for sample collection, circulating proteome may serve as an important source for uncovering biomarkers that could be useful for CRC risk assessment and identifying high-risk populations for CRC screening. Nevertheless, studies implementing proteomics technologies, which enables an agnostic scan of thousands of proteins, are still limited and the majority of the studies focused on developing diagnostic biomarkers for early detection of CRC [15,16]. Their findings, however, are unlikely to be translated into novel risk assessment tools since, by nature, diagnostic biomarkers are not optimized for predicting future disease. To date, only a few studies have used prediagnostic blood samples to conduct a systematic search for risk biomarkers of CRC using proteomics technologies [17]. Of note, prior studies were predominantly carried out in populations of European ancestry. Here, we conducted a two-stage nested case-control study within the Shanghai Women’s Health Study (SWHS) to screen and validate potential circulating protein markers for CRC risk in East Asians. 

## 2. Methods and Materials

### 2.1. Study Design, Population, and Data

The SWHS launched in 1996 to 2000 in Shanghai, China. The information has been detailed elsewhere [18]. Briefly, SWHS enrolled 74,947 women aged 40–70 years from 7 urban neighborhoods in Shanghai. All participants completed an in-person interview using structured questionnaires. Of the study participants, 56,831 (75.8%) provided a 10 mL blood sample at the baseline recruitment. All blood samples were kept at 4 °C during transportation. Within 6 h after the collection, blood samples were processed, and plasma specimens were separated and aliquoted for long-term storage at −80 °C.

Incident cancer cases were identified by annual linkages to Shanghai Cancer Registry and Shanghai Vital Statistic Unit as well as in-person follow-up surveys conducted every 2 to 6 years. Cancer diagnosis was confirmed by review of medical records. In the present study, we randomly selected 100 cases for the discovery phase from all eligible incident CRC cases reported in the SWHS according to the following criteria: (1) provided blood samples and (2) the interval between age at diagnosis and age at blood collection >1 year and <10 years. In the validation phase, 60 CRC cases were randomly selected to resemble the cases in the discovery phase. For each case, one cancer-free control was selected by incidence-density sampling method and matched by menopausal status at sample collection (yes/no), time of sample collection (morning or afternoon), antibiotic use in the past week (yes/no), age at sample collection (within 2 years), date at sample collection (within 30 days) and time interval since last meal (within 2 h).

This study was approved by the institutional review boards of all institutes involved. Each participant provided written informed consent at enrollment.

### 2.2. Laboratory Methods

A total of 1104 proteins were characterized in the discovery stage using the proximity extension assays (PEA) with 12 of the Olink Proseek panels (CAM, CRE, CVDⅡ, CVDⅢ, DEV, INF, IRE, MET, NEU, NEX, ODA, ONCⅡ). Detailed information about the used panels is available online (www.olink.com, accessed on 1 May 2022). Briefly, the PEA assay is an affinity-based assay to evaluate the abundance levels of circulating proteins. For each target protein, a pair of oligonucleotide-labeled antibody binding probes were designed. When the two probes are in proximity, a PCR target sequence is formed by a proximity-dependent DNA polymerization event. The sequence is then subsequently detected and quantified using standard real-time PCR. The final assay readout was presented in the Normalized Protein Expression (NPX) values, which were Ct values converted and expressed in log2-scale. A high NPX value corresponds to a high protein concentration. Internal controls were added to each sample for quality control (QC) purpose during the protein profiling. The internal controls for the incubation, extension, and amplification step, were spiked into each sample for each dilution and panel. Samples that deviated less than 0.3 NPX from the median of all samples in one of two control assays for incubation and amplification passed the QC. Additionally, samples representing external, negative, and inter-plate controls were included in each sample plate. Each assay has an experimentally determined lower limit of detection (LOD), which is three standard deviations (SDs) above the noise level. All assay values below LOD were replaced with the defined LOD-value. In the discovery phase of the current study, two cases failed in the QC procedures and the corresponding controls were excluded from downstream analyses. Thirty-six proteins were contained in more than one Olink panel. We removed the duplicates that have a higher coefficient of variation (CV) in our QC samples. We further excluded 98 proteins with more than 30% of CV and retained 970 proteins for further analysis. The CVs for each protein are displayed in Supplementary Table S1. Principal components analysis (PCA) was performed and Supplementary Figure S1 shows the first two PC of proteomics data generated from the pooled sample controls and actual cohort samples. The well-clustered pooled controls suggested that the measurement of actual cohort samples should be reliable.

Plasma protein levels in the validation set were profiled by the Olink Explore 1536 assay. This assay uses PEA technology coupled to readout metrology based on Next Generation Sequencing (NGS). Counts of known sequences were converted to NPX units by quality control and normalization process. For the present study, we focused on the proteins that showed a significant association with CRC in the discovery phase (*p_discovery_* < 0.05) and performed the downstream analyses. In the validation, all samples and protein markers passed QC. The inter-assay CVs for the identified proteins in the validation set of samples are shown in Supplementary Table S2.

### 2.3. Statistical Analysis

To assess the associations between circulating proteins and CRC risk, we first treated circulating proteins as continuous variables. Conditional logistic regression was used to calculate the odds ratio (OR) and 95% confidence intervals (CIs). Given that most risk factors varied little between cases and controls in our study [18], we minimally adjusted for age, educational level, and body mass index (BMI, kg/m^2^) for proteins that passed the QC procedures. ORs were calculated with respect to 1 SD change in protein levels. For proteins with a high proportion of LOD (>50%, *n*_protein_ = 59) in the discovery set, we dichotomized them as follows: individuals with LOD values were grouped into the category of low abundance and the remaining were classified as the high abundance group. In the validation set, individuals were classified into two groups according to the median levels of the corresponding protein in the controls (since the sensitivity of the platform has been significantly improved by coupling with NGS technology, no protein in the validation required extensive imputation with LOD). Subjects with a low protein abundance served as the reference group in the association test for the protein. The multivariable adjusted ORs (per 1-SD change) and 95% CI from the discovery and validation phases were combined using fixed-effects or random-effects meta-analysis implemented in the “metafor” package (“rma” function) to derive pooled estimates. Sensitivity analyses were performed with the exclusion of cases diagnosed within the first two years of follow-up. Pairwise Spearman’s rank correlations with adjustment of age were performed separately in the discovery and validation set. An elastic net regression (alpha = 0.5) with a 5-fold cross-validation procedure was performed to construct a protein score for CRC risk in the discovery set. The score was calculated as the weighted sum of the selected proteins with weights equal to coefficients from the elastic net regression. The association of CRC risk with the score either in a continuous form with respect to 1-SD change in its original scale or dichotomized based on the distribution among controls was then evaluated. The same procedures were repeated in the validation set with the same coefficients brought from the discovery set. All statistical analyses were performed using R version 4.1.1 (R Foundation for Statistical Computing, Vienna, Austria). A nominal two-sided *p* value < 0.05 was considered a significance threshold in both discovery and validation sets.

## 3. Results

**Figure 1:**
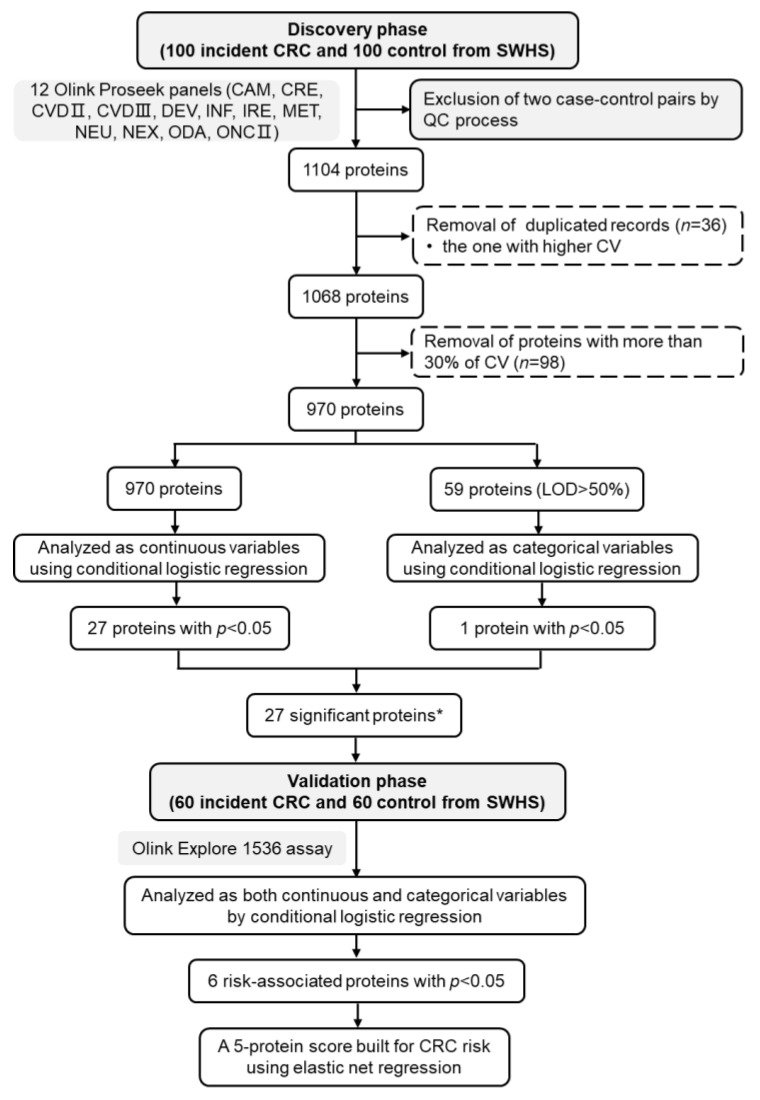
The workflow of the present study. * The protein identified in the analysis in which proteins were treated as categorical variables was already identified by the analysis in which proteins were treated as continuous variables in the regression models. Abbreviations: CV, coefficient of variation; LOD, lower limit of detection; SWHS, Shanghai Women’s Health Study.

A summary flow diagram of this study is shown in Figure 1. Host characteristics of study participants are presented in Table 1. In the discovery phase, controls were on average slightly younger than cases (*p* = 0.021), whereas there were no significant differences in other sociodemographic characteristics, lifestyles, and health status between the two groups in both sets (*p* > 0.05) (Table 1). The distributions of host characteristics were nearly identical between the discovery and validation sets (Supplementary Table S3).

In the discovery phase, 27 proteins were nominally significantly associated with CRC risk in the multivariable models (Table 2). Among them, 22 markers were positively associated with CRC risk, whereas 5 showed an inverse relationship. In the validation phase, 6 of the 27 identified proteins retained a nominally significant association with CRC risk (Table 2). The pooled ORs by meta-analysis corresponding to 1 SD increase of protein levels were 1.54 (95% CI: 1.15–2.06) for B-cell antigen receptor complex-associated protein beta chain (CD79B), 1.71 (95% CI: 1.24–2.34) for epithelial discoidin domain-containing receptor 1 (DDR1), 2.04 (95% CI: 1.39–3.01) for ephrin-A4 (EFNA4),1.54 (95% CI: 1.16–2.02) for leucine-rich repeat transmembrane protein FLRT2 (FLRT2), 2.09 (95% CI: 1.47–2.98) for leukotriene A-4 hydrolase (LTA4H), and 1.88 (95% CI: 1.35–2.62) for natural cytotoxicity triggering receptor 1 (NCR1). Additionally, suggestive associations with CRC risk were found for 13 proteins in the meta-analysis (*p_meta_* < 0.05) (Table 2).

**Figure 2:**
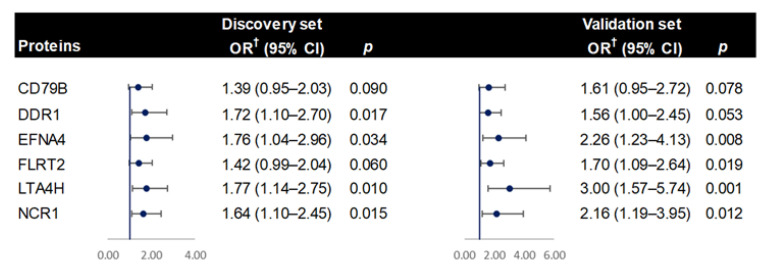
Results of the sensitivity analyses conducted for the identified six protein markers in both the discovery and validation phases. ^†^ Odds radio (OR) was calculated with respect to 1-SD increase in protein level.

For proteins having a high proportion of LOD values (>50%), we additionally treated them as dichotomized variables in the analysis. Proteins with a significant association with CRC are presented in Supplementary Table S4. The elevated levels of LTA4H were found significantly correlated with an increased risk of CRC with an OR of 2.55 (95% CI: 1.30–5.02) and 7.40 (95% CI: 2.11–25.93) in the discovery and validation set, respectively. No additional proteins were identified through this analysis. Additional sensitivity analyses showed consistent associations for all identified proteins after excluding cases diagnosed within the first two years of follow-up, except for CD79B, as its association was attenuated in both discovery and validation sets (Figure 2).

As shown in Supplementary Figure S2, significant correlations were found among the identified proteins (*p* < 0.05), of which the correlation coefficients ranged between 0.18 and 0.57 in the discovery set. These correlations were all replicated in the validation set. We subsequently performed elastic net regression to build a multi-protein biomarker score based on the six proteins identified using our discovery data. The β coefficients for CD79B (β = 0.122), DDR1 (β = 0.110), FLRT2 (β = 0.046), LTA4H (β = 0.340), and NCR1 (β = 0.141) remained non-zero in the penalized regression and hence were selected to build the score in both discovery and validation sets. A significant association was observed between the protein score and CRC risk in both sets (continuous form, 1-SD increase, discovery: OR = 2.46, 95% CI: 1.53–3.95; validation: OR = 4.16, 95% CI: 1.92–8.99) (Table 3). Similarly, for the participants with a high dichotomized protein score, the ORs for CRC risk were 2.87 (95% CI: 1.38–5.95) and 4.88 (95% CI: 1.76–13.50) in the discovery and validation set, respectively (Table 3). Finally, no biological protein-protein interaction was found by inquiring with the STRING database for the identified proteins (https://string-db.org/, accessed on 1 May 2022).

## 4. Discussion

We performed a two-staged nested case-control study within the SWHS to identify potential novel protein biomarkers for CRC risk. In the discovery set, we found 27 circulating proteins with a nominally significant association. Six of the 27 proteins, including CD79B, DDR1, EFNA4, FLRT2, LTA4H, and NCR1, were validated in the validation phase of the study.

Different types of molecules, such as microRNAs, autoantibodies, and metabolites, have been assessed for their value in CRC risk assessment and screening [19,20]. However, a limited number of reported biomarkers have been validated and only few (may) have clinical utilities, such as carcinoembryonic antigen (CEA) [19,21,22]. Numerous studies have been conducted to discover potential non-invasive biomarkers for early diagnosis/detection of CRC by investigating circulating proteome in post-diagnosis blood samples collected from CRC patients [15,23]. The proteins reported in these studies, however, may not be involved or detectable in the pre-carcinogenic or early carcinogenic states, and thus may not be used as risk-assessment tools in general or high-risk populations. Evaluation of pre-diagnostic samples, on the other hand, may optimize the chance of finding promising candidates for effective risk assessment and personalized screening [24,25]. For instance, one study by Harlid and colleagues, examined ~160 proteins and other biomarkers and found that fibroblast growth factor 21 and pancreatic prohormone were associated with risk of colon and rectal cancer, respectively [17]. Another case-cohort study conducted in a Japanese population analyzed 62 inflammatory factors and identified several chemokines for CRC risk but lacked validation [26]. Although these protein markers were not replicated in the current study [17,26], the discrepancies may result from the differences in study populations, proteomics platforms, or analytical approaches.

Here, we found that circulating levels of LTA4H showed a strong association with CRC risk. LTA4H is a bifunctional zinc-dependent enzyme that catalyzes the final rate-limiting step in the biosynthesis of leukotriene B4, a potent inducer of neutrophil, macrophage, and T lymphocyte chemotaxis [27,28]. LTA4H also possesses aminopeptidase activity, which is assumed to participate in the processing of peptides related to inflammation and host defense [29,30]. An in vitro study showed that knockdown of LTA4H or treatment with its inhibitor could attenuate proliferation and colony formation of CRC cells [31,32]. Our results support the hypothesis that LTA4H may play a critical role in the development of CRC.

Our findings for several other circulating proteins were also in line with results from prior research in CRC tumors and/or animal models. EFNA4 belongs to the ephrin family, which anchors to the membrane via glycosylphosphatidylinositol linkage to mediate cancer cell growth, migration, and invasion [33,34,35]. By characterizing proteins secreted by colon tumor cells, a previous study found that EFNA4 was abundant in the LIM1215 cell culture media and interstitial fluid, supporting its potential role in CRC biology [36]. DDR1 is a member of the receptor tyrosine kinases subfamily that binds to collagen and acts as a central extracellular matrix sensor for cell adhesion [37]. Hu et al. found that DDR1 can promote CRC cell invasion and metastatic behavior in nude mice [38]. It is proposed that the activity of DDR1 can promote β-catenin oncogenic activity to sustain tumor cell migration, survival, and renewal [39,40]. NCR1 is a pivotal member of the NCR family that was expressed on both resting and activated NK cells. It was reported to participate in the process of influenza infection, diabetes, as well as tumorigenesis [41,42]. However, further research is still warranted for elucidating its role in CRC development.

There is limited knowledge on potential biological mechanisms to explain the observed associations for some of the proteins identified in this study. For example, CD79B is expressed exclusively on mature B cells and functions as the main signaling component of B cell antigen receptor complexes [43]. Inconsistent with our findings, a prior study showed that expression of CD79B is downregulated in tissue samples of early age-onset CRC cases [44]. Further, as a member of the FLRT family, FLRT2 functions as an adhesion molecule by interacting with fibronectin in either a repulsive or adhesive manner [45]. It is reported that downregulated expression of FLRT2 was observed in CRC tumor samples compared to matched normal epithelial mucosa [46]. Causes for these discrepancies are likely to be multifactorial. Study design and heterogeneity between different study populations may contribute to the inconsistent findings. It is also possible that some of our results were affected by the state of the disease. This may explain why in our study the observed significant association of CD79B was diminished when cases diagnosed within the first two years of follow-up were excluded from the analysis. Such changes may reflect the impact of a small sample size or imply that CD79B could be a disease biomarker rather than a risk biomarker. Nonetheless, results from the sensitivity analysis suggest the robustness for the remaining identified proteins. Finally, a few additional proteins with a suggestive association were found. Some of them, such as dipeptidyl peptidase 1, serine protease inhibitor Kazal-type 5, ephrin type-B receptor 6 and transferrin receptor protein 1, were previously reported to be involved in colorectal carcinogenesis [47,48,49,50].

The major strengths of the present study include the rigorous two-stage design and the use of prospectively collected epidemiological data and prediagnostic samples; thus, the impact of recall bias and reverse causation is minimized. Additionally, this is the first study that evaluated over 1000 circulating proteins in prediagnostic blood samples for their associations with CRC risk in Asians, an underrepresented population in biomarker research for CRC risk.

Despite the strengths, we also recognize several limitations in our study. First, the associations that we found were relatively modest and not significant after correction for multiple comparisons. However, our rigorous discovery-validation design has mitigated the concern for false positive findings. The relatively small sample size of our datasets may lead to a reduced power, therefore false negatives may be another concern. In addition, since all participants in the current study were Chinese women, our findings may not be generalizable to other populations. Further large-scale investigations are required to verify our findings in a similar population, as well as in other racial/ethnic groups. Finally, mechanistic experiments are warranted to uncover the biological mechanisms involved in the observed associations. Investigations into establishing the utility of the validated biomarkers in CRC risk-assessment and screening are also critically needed.

## 5. Conclusions

We conducted a prospective proteomics investigation to identify potential protein biomarkers for CRC risk in East Asian women. Our findings provide novel insights into the etiology of CRC and may facilitate its risk assessment. However, further research is required to further validate our findings and uncover the underlying biological mechanisms involved in colorectal carcinogenesis.

## Figures and Tables

**Table 1:** Baseline characteristics of study participants in the discovery and validation sets.

Characteristics	Discovery, No. (%)			Validation, No. (%)		
	Case (*n* = 98)	Control (*n* = 98)	*p*	Case (*n* = 60)	Control (*n* = 60)	*p*
Age at blood draw, mean (SD), y	59.2 (8.8)	58.92 (8.7)	0.021	60.1 (8.7)	60.1 (8.7)	0.998
BMI, mean (SD), kg/m^2^	24.4 (3.0)	24.9 (3.5)	0.295	25.2 (4.0)	25.0 (3.4)	0.806
WHR, mean (SD)	0.8 (0.1)	0.8 (0.1)	0.409	0.8 (0.1)	0.8 (0.1)	0.844
Family income, %						
<10,000 RMB	23 (23.5)	19 (19.4)	0.922	18 (30.0)	18 (30.0)	0.765
10,000 RMB-	40 (40.8)	42 (42.9)		22 (36.7)	21 (35.0)	
20,000 RMB-	20 (20.4)	21 (21.4)		17 (28.3)	15 (25.0)	
≥30,000 RMB	15 (15.3)	16 (16.3)		3 (5.0)	6 (10.0)	
Educational attainment, %						
≤Elementary school	34 (34.7)	43 (43.9)	0.532	27 (45.0)	25 (41.7)	0.611
Middle school	29 (29.6)	24 (24.5)		16 (26.7)	21 (35.0)	
High school	22(22.4)	17 (17.3)		10 (16.7)	6 (10.0)	
≥College	13 (13.3)	14 (14.3)		7 (11. 7)	8 (13.3)	
Physical activity, mean (SD), MET-hrs/day/yrs	0.9 (1.4)	1.0 (1.5)	0.759	1.0 (1.4)	1.0 (2.0)	0.876
Family history of adenomatous polyposis of colorectum, %	1 (1.0)	0 (0.0)	1.000	0 (0.0)	0 (0.0)	1.000
Family history of colorectal cancer, %	3 (3.1)	1 (1.0)	0.613	2 (3.3)	0 (0.0)	0.476
Current aspirin use, %	4 (4.1)	3 (3.1)	1.000	3 (5.0)	2 (3.3)	1.000
Current peptic ulcer medication use, %	4 (4.1)	3 (3.1)	1.000	2 (3.3)	1 (1.7)	1.000
Ulcerative colitis, %	1 (1.0)	0 (0.0)	1.000	0 (0.0)	1 (1.7)	1.000
Diabetes, %	7 (7.1)	11 (11.2)	0.458	4 (6. 7)	5 (8.3)	1.000
Colorectal polyp, %	2 (2.0)	1 (1.0)	1.000	1 (1.7)	1 (1.7)	1.000
Total energy, mean (SD), Kcal	1677.1 (426.6)	1673.2 (398.3)	0.946	1613.8 (403.0)	1642.6 (395.8)	0.693
Red meat, mean (SD), g/day/1000 Kcal	30.0 (18.9)	29.62 (22.1)	0.897	25.2 (16.9)	26.1 (17.6)	0.773
Fat, mean (SD), g/day/1000 Kcal	17.6 (5.7)	17.77 (6.5)	0.858	15.7 (6.0)	15.8 (4.9)	0.922
Fruit, mean (SD), g/day/1000 Kcal	138.1 (95.4)	150.0(93.5)	0.381	107.7 (87.9)	130.5 (79.4)	0.140
Vegetable, mean (SD), g/data/1000 Kcal	171.7 (80.3)	196.7 (106.6)	0.066	165.3 (82.0)	180.0 (86.6)	0.341

Abbreviation: BMI, body mass index; WHR, waist-to-hip ratio.

**Table 2:** Associations between selected protein markers and colorectal cancer risk.

Proteins	Discovery		Validation		Meta-Analysis	
	OR (95% CI) ^a^	*p*	OR (95% CI) ^a^	*p*	OR (95% CI) ^a^	*p*
ADAM22	1.50 (1.01–2.22)	0.046	1.37 (0.85–2.22)	0.196	1.44 (1.06–1.96)	0.018
AGR3	0.72 (0.52–1.00)	0.047	1.38 (0.91–2.09)	0.132	0.98 (0.52–1.86)	0.953
Beta-NGF	1.70 (1.07–2.69)	0.024	0.85 (0.55–1.30)	0.447	1.19 (0.60–2.36)	0.611
CANT1	1.63 (1.05–2.52)	0.028	1.27 (0.86–1.88)	0.225	1.42 (1.06–1.90)	0.018
CASP-8	1.47 (1.04–2.08)	0.030	1.62 (0.96–2.74)	0.072	1.51 (1.13–2.02)	0.005
CD79B	1.47 (1.02–2.13)	0.039	1.65 (1.03–2.66)	0.038	1.54 (1.15–2.06)	0.004
CDH17	0.71 (0.51–0.97)	0.034	1.19 (0.79–1.81)	0.403	0.90 (0.54–1.51)	0.695
CLM-1	1.55 (1.01–2.37)	0.044	1.37 (0.89–2.11)	0.158	1.46 (1.08–1.98)	0.015
CRTAM	1.48 (1.04–2.13)	0.032	1.20 (0.75–1.91)	0.449	1.37 (1.03–1.82)	0.030
CTSC	1.51 (1.07–2.13)	0.019	0.79 (0.11–5.87)	0.818	1.48 (1.05–2.08)	0.023
DDR1	1.73 (1.11–2.70)	0.015	1.68 (1.07–2.64)	0.026	1.71 (1.24–2.34)	0.001
EFNA4	1.86 (1.11–3.14)	0.019	2.29 (1.28–4.09)	0.005	2.04 (1.39–3.01)	3.11 × 10^−4^
EPHB6	1.85 (1.20–2.85)	0.005	1.33 (0.87–2.05)	0.190	1.57 (1.16–2.13)	0.004
FABP9	0.68 (0.47–0.98)	0.041	1.35 (0.88–2.07)	0.167	0.95 (0.48–1.87)	0.879
FLRT2	1.44 (1.00–2.08)	0.049	1.67 (1.09–2.54)	0.018	1.54 (1.16–2.02)	0.002
HSP-27	0.69 (0.50–0.95)	0.025	1.52 (0.89–2.58)	0.127	0.99 (0.46–2.15)	0.983
HSP90B1	1.71 (1.18–2.48)	0.005	0.77 (0.48–1.23)	0.274	1.16 (0.53–2.54)	0.707
IL-6RA	1.50 (1.04–2.17)	0.028	1.27 (0.85–1.91)	0.246	1.40 (1.06–1.83)	0.016
LTA4H	1.78 (1.16–2.74)	0.008	2.93 (1.57–5.46)	0.001	2.09 (1.47–2.98)	4.44 × 10^−5^
MATN3	1.58 (1.09–2.29)	0.017	1.09 (0.73–1.65)	0.669	1.34 (1.01–1.76)	0.039
NCR1	1.70 (1.14–2.54)	0.009	2.34 (1.29–4.23)	0.005	1.88 (1.35–2.62)	1.90 × 10^−4^
SLAMF8	1.43 (1.00–2.02)	0.047	1.35 (0.80–2.28)	0.267	1.40 (1.05–1.88)	0.024
SPINK5	1.55 (1.08–2.23)	0.018	1.54 (0.98–2.42)	0.064	1.55 (1.16–2.05)	0.003
TR	1.52 (1.04–2.23)	0.031	1.35 (0.88–2.06)	0.167	1.44 (1.09–1.91)	0.011
TRANCE	1.52 (1.06–2.17)	0.022	1.11 (0.76–1.63)	0.580	1.31 (1.01–1.70)	0.040
UNC5C ^b^	1.91 (1.18–3.08)	0.008	-	-	-	-
WAS	0.71 (0.52–0.97)	0.034	0.94 (0.64–1.37)	0.731	0.79 (0.62–1.01)	0.065

^a^ Odds radio (OR) was calculated with respect to 1-SD increase in circulating protein levels, obtained from the conditional logistic regression with the adjustment of age, educational level, and BMI. ^b^ UNC5C was not included in the Olink Explore 1536 assay in validation set.

**Table 3:** The associations between the derived protein risk score and colorectal cancer risk.

5-Protein Score	Discovery		Validation	
	OR (95% CI)	*p*	OR (95% CI)	*p*
Continuous score ^a^	2.46 (1.53–3.95)	1.97 × 10^−4^	4.16 (1.92–8.99)	2.97 × 10^−4^
Categorical score ^b^				
Low level	Ref		Ref	
High level	2.87 (1.38–5.95)	0.005	4.88 (1.76–13.50)	2.27 × 10^−4^

^a^ Odds radio (OR) was calculated with respect to 1-SD increase in score. It was obtained from the conditional logistic regression with the adjustment of age, educational level, and BMI. ^b^ Dichotomous cutoffs were determined by the median of protein score among controls. In the discovery phase, 5-protein scores for dichotomous cutoffs were <7.0252 for the low group (*n*_case_ = 29 and *n*_control_ = 49), and ≥7.0252 for the high group (*n*_case_ = 69, *n*_control_ = 49). In the validation phase, 5-protein scores dichotomous cutoffs were <1.4436 for the low group (*n*_case_ = 12 and *n*_control_ = 30), and ≥1.4436 for the high group (*n*_case_ = 48 and *n*_control_ = 30).

## Data Availability

The data presented in this study are available on request from the corresponding author.

## Supplementary Materials

The following supporting information can be downloaded at: https://www.mdpi.com/article/10.3390/cancers14133261/s1. Figure S1: Scatter plot for the first two principal components of the quality control and cohort samples; Figure S2: Age-adjusted Spearman correlation coefficients of 6 risk-associated proteins; Table S1: The inter-assay coefficient of variation for all proteins analyzed in the discovery set; Table S2: The inter-assay coefficient of variation for the 27 proteins tested in the validation set; Table S3: The comparison of host characteristics between the individuals involved in the discovery and validation set; Table S4: The associations between CRC risk and selected dichotomized protein markers.
