# Supplementary material for: Prospective Proteomic Study Identifies Potential Circulating Protein Biomarkers for Colorectal Cancer Risk
Source: Cancers (Basel). 2022 Jul 3;14(13):3261. doi: 10.3390/cancers14133261 (PMC9265260; doi:10.3390/cancers14133261)
Supplement: Supplementary file 1 [file cancers-14-03261-s001.zip › cancers-1763466-supplementary.pdf]

Supplemental Content

Supplementary Figure S1. Scatter plot for the first two principal components of the quality control and cohort samples.

Supplementary Figure S2. Age-adjusted Spearman correlation coefficients of 6 risk-associated proteins.

*****$P<0.05$

Supplementary Table S1. The inter-assay coefficient of variation for all proteins analyzed in the discovery set.

Supplementary Table S2. The inter-assay coefficient of variation for the 27 proteins tested in the validation set.

Supplementary Table S3. The comparison of host characteristics between the individuals involved in the discovery and validation set.

Supplementary Table S4. The associations between CRC risk and selected dichotomized protein markers.

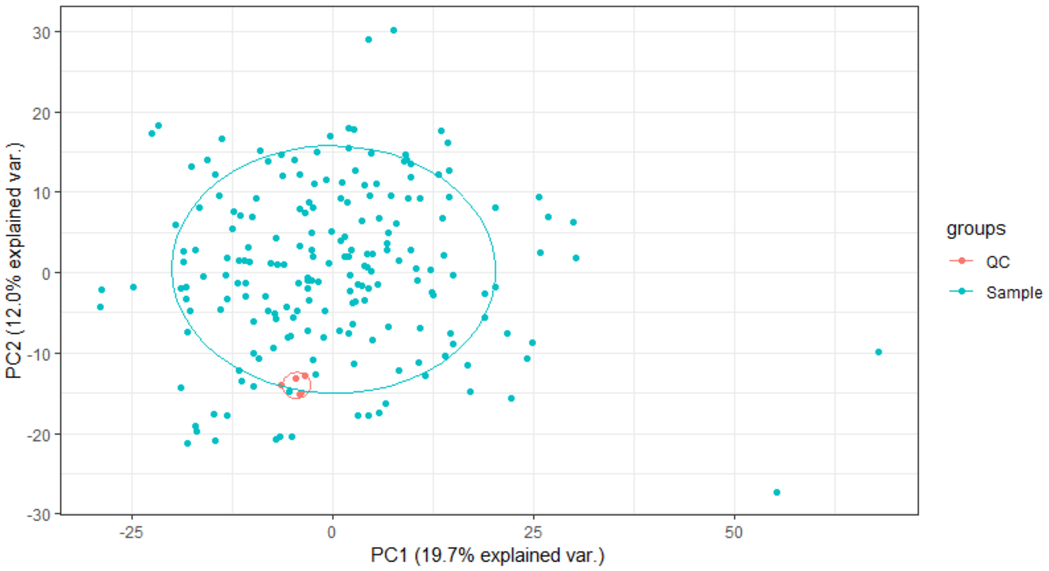

Figure S1. Scatter plot for the first two principal components of the quality control and cohort samples.

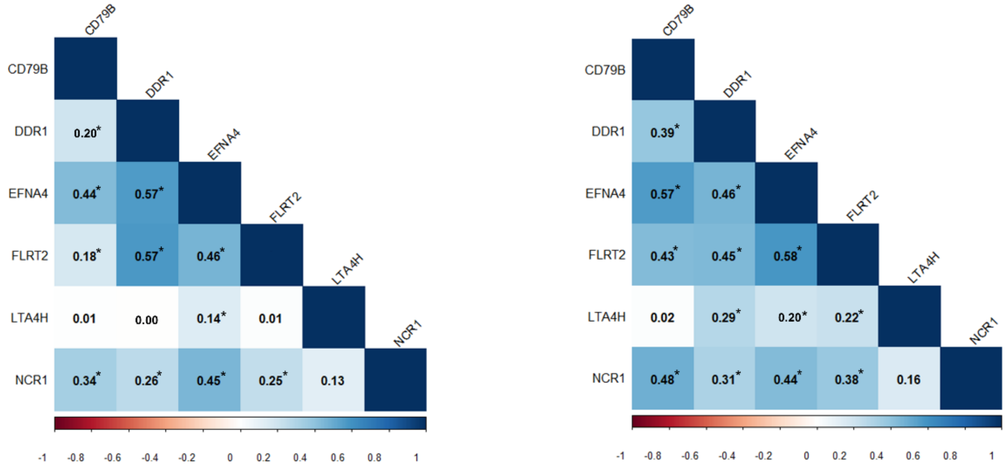

Figure S2. Age-adjusted Spearman correlation coefficients of 6 risk-associated proteins. **P*<0.05

Table S1. The inter-assay coefficient of variation for all proteins analyzed in the discovery set.

**Proteins**	**Gene**	**CV**
Alanyl-tRNA editing protein Aarsd1	AARSD1	15.73
Protein ABHD14B	ABHD14B	21.87
Tyrosine-protein kinase ABL1	ABL1	8.37
Aggrecan core protein	ACAN	7.59
Angiotensin-converting enzyme 2	ACE2	6.41
Lysophosphatidic acid phosphatase type 6	ACP6	14.05
Adenosine Deaminase	ADA	5.36
Disintegrin and metalloproteinase domain-containing protein 15	ADAM15	15.49

Disintegrin and metalloproteinase domain-containing protein 22	ADAM 22	9.75
Disintegrin and metalloproteinase domain-containing protein 23	ADAM 23	13.56
Disintegrin and metalloproteinase domain-containing protein 8	ADAM8	7.23
A disintegrin and metalloproteinase with thrombospondin motifs 13	ADAM-TS13	3.00
A disintegrin and metalloproteinase with thrombospondin motifs 15	ADAM-TS 15	9.62
Adhesion G protein-coupled receptor E2	ADGRE2	12.68
Adhesion G-protein coupled receptor G1	ADGRG1	8.25
Adhesion G-protein coupled receptor G2	ADGRG2	11.30
ADM	ADM	4.59
Anterior gradient protein 2 homolog	AGR2	15.34
Anterior gradient protein 3	AGR3	17.02
Agouti-related protein	AGRP	9.05
Adenosylhomocysteinase	AHCY	10.52
Apoptosis-inducing factor 1, mitochondrial	AIFM1	26.97
Proline-rich AKT1 substrate 1	AKT1S1	16.29
CD166 antigen	ALCAM	8.14
Retinal dehydrogenase 1	ALDH1A1	9.96
Aldehyde dehydrogenase, dimeric NADP-preferring	ALDH3A1	10.49
Alpha-2-macroglobulin receptor-associated protein	Alpha-2-MRAP	12.87
Protein AMBP	AMBP	4.13
Amphoterin-induced protein 2	AMIGO2	13.62
Protein amnionless	AMN	16.56
Angiogenin	ANG	29.45
Angiopoietin-2	ANGPT2	6.73
Angiopoietin-related protein 1	ANGPTL1	6.70
Angiopoietin-related protein 3	ANGPTL3	32.27
Angiopoietin-related protein 4	ANGPTL4	11.12
Angiopoietin-related protein 7	ANGPTL7	5.43
Annexin A1	ANXA1	7.55
Annexin A10	ANXA10	11.12
Annexin A11	ANXA11	21.63
Annexin A4	ANXA4	0.00
Amiloride-sensitive amine oxidase (copper-containing)	AOC1	6.73
Membrane primary amine oxidase	AOC3	33.84
Amyloid beta A4 precursor protein-binding family B member 1-interacting protein	APBB1IP	18.54
DNA-(apurinic or apyrimidinic site) lyase	APEX1	10.19
Amyloid-like protein 1	APLP1	7.88
Aminopeptidase N	AP-N	7.37
Apolipoprotein M	APOM	39.66
Amyloid beta A4 protein	APP	9.29

Amphiregulin	AREG	7.17
Arginase-1	ARG1	101.17
Rho GTPase-activating protein 1	ARHGAP1	15.48
Rho guanine nucleotide exchange factor 12	ARHGEF12	20.69
Aryl hydrocarbon receptor nuclear translocator	ARNT	0.00
Arylsulfatase A	ARSA	11.93
Arylsulfatase B	ARSB	9.29
Artemin	ARTN	3.34
Asialoglycoprotein receptor 1	ASGR1	14.62
Cysteine protease ATG4A	ATG4A	11.52
Renin receptor	ATP6AP2	17.86
V-type proton ATPase subunit F	ATP6V1F	16.64
Axin-1	AXIN1	18.98
Tyrosine-protein kinase receptor UFO	AXL	9.96
Azurocidin	AZU1	51.78
Beta-1,4-galactosyltransferase 1	B4GALT1	4.87
Beta-1,4-glucuronyltransferase 1	B4GAT1	10.61
Transcription regulator protein BACH1	BACH1	8.13
Large proline-rich protein BAG6	BAG6	26.77
BMP and activin membrane-bound inhibitor homolog	BAMBI	25.34
B-cell scaffold protein with ankyrin repeats	BANK1	13.48
Basal cell adhesion molecule	BCAM	6.10
Brevican core protein	BCAN	10.12
Bcl-2-like protein 11 isoform BimL	BCL2L11	13.26
Breakpoint cluster region protein	BCR	24.40
Beta-nerve growth factor	Beta-NGF	1.25
Biglycan	BGN	5.87
BH3-interacting domain death agonist	BID	26.45
Baculoviral IAP repeat-containing protein 2	BIRC2	0.00
Bleomycin hydrolase	BLM hydrolase	9.04
Flavin reductase NADPH	BLVRB	18.26
Bone morphogenetic protein 4	BMP-4	5.87
Bone morphogenetic protein 6	BMP-6	23.41
Natriuretic peptides B	BNP	0.00
Brother of CDO	BOC	6.00
ADP-ribosyl cyclase/cyclic ADP-ribose hydrolase 2	BST1	6.39
Bone marrow stromal antigen 2	BST2	14.39
Probetacellulin	BTC	33.56
Butyrophilin subfamily 3 member A2	BTN3A2	11.80
Complement C1q tumor necrosis factor-related protein 1	C1QTNF1	42.69
Complement C2	C2	36.58

Carbonic anhydrase 1	CA1	33.11
Carbonic anhydrase 12	CA12	12.46
Carbonic anhydrase 13	CA13	5.11
Carbonic anhydrase 14	CA14	15.47
Carbonic anhydrase 2	CA2	15.50
Carbonic anhydrase 3	CA3	27.77
Carbonic anhydrase 4	CA4	26.88
Carbonic anhydrase 5A, mitochondrial	CA5A	7.93
Carbonic anhydrase 6	CA6	9.93
Cell adhesion molecule 3	CADM3	15.66
Carbonic anhydrase IX	CAIX	13.25
Calcitonin	CALCA	21.05
Calreticulin	CALR	25.41
Calcium/calmodulin-dependent protein kinase kinase 1	CAMKK1	7.54
Soluble calcium-activated nucleotidase 1	CANT1	6.20
Macrophage-capping protein	CAPG	26.22
Calcium-regulated heat-stable protein 1	CARHSP1	17.54
Caspase-3	CASP-3	39.64
Caspase-8	CASP-8	12.41
E3 ubiquitin-protein ligase CBL	CBL	22.01
Coiled-coil domain-containing protein 80	CCDC80	11.92
Eotaxin	CCL11	6.28
C-C motif chemokine 14	CCL14	29.93
C-C motif chemokine 15	CCL15	14.56
C-C motif chemokine 16	CCL16	12.38
C-C motif chemokine 17	CCL17	12.23
C-C motif chemokine 18	CCL18	32.70
C-C motif chemokine 19	CCL19	12.04
C-C motif chemokine 20	CCL20	13.52
C-C motif chemokine 21	CCL21	9.48
C-C motif chemokine 23	CCL23	14.96
C-C motif chemokine 24	CCL24	10.89
C-C motif chemokine 25	CCL25	12.51
C-C motif chemokine 27	CCL27	25.70
C-C motif chemokine 28	CCL28	13.22
C-C motif chemokine 3	CCL3	16.04
C-C motif chemokine 4	CCL4	12.81
C-C motif chemokine 5	CCL5	29.07
CD109 antigen	CD109	15.39
CD160 antigen	CD160	9.39
Scavenger receptor cysteine-rich type 1 protein M130	CD163	9.18

Sialomucin core protein 24	CD164	10.80
CD177 antigen	CD177	16.55
T-cell surface glycoprotein CD1c	CD1C	4.78
OX-2 membrane glycoprotein	CD200	10.18
Cell surface glycoprotein CD200 receptor 1	CD200R1	5.83
C-type lectin domain family 4 member K	CD207	10.19
CD209 antigen	CD209	7.16
Natural killer cell receptor 2B4	CD244	11.44
CD27 antigen	CD27	7.50
T-cell-specific surface glycoprotein CD28	CD28	0.00
CD2-associated protein	CD2AP	105.21
CMRF35-like molecule 9	CD300LG	6.02
CD302 antigen	CD302	15.02
Myeloid cell surface antigen CD33	CD33	17.57
ADP-ribosyl cyclase/cyclic ADP-ribose hydrolase 1	CD38	11.34
T-cell surface glycoprotein CD4	CD4	3.63
CD40L receptor	CD40	13.29
CD40 ligand	CD40-L	24.21
Membrane cofactor protein	CD46	34.41
CD48 antigen	CD48	5.53
T-cell surface glycoprotein CD5	CD5	11.02
Lymphocyte function-associated antigen 3	CD58	11.94
CD59 glycoprotein	CD59	18.90
T cell surface glycoprotein CD6 isoform	CD6	20.93
CD63 antigen	CD63	19.42
Early activation antigen CD69	CD69	20.29
CD70 antigen	CD70	14.36
HLA class II histocompatibility antigen gamma chain	CD74	14.08
B-cell antigen receptor complex-associated protein beta chain	CD79B	5.25
CD83 antigen	CD83	7.79
SLAM family member 5	CD84	4.31
T-cell surface glycoprotein CD8 alpha chain	CD8A	16.76
Complement component C1q receptor	CD93	8.46
CD97 antigen	CD97	13.67
CD99 antigen-like protein 2	CD99L2	9.82
CUB domain-containing protein 1	CDCP1	13.85
Cadherin-1	CDH1	34.85
Cadherin-15	CDH15	30.05
Cadherin-17	CDH17	17.18
Cadherin-2	CDH2	4.35
Cadherin-3	CDH3	10.11

Cadherin-5	CDH5	9.54
Cadherin-6	CDH6	7.72
Cadherin-related family member 5	CDHR5	6.53
Cyclin-dependent kinase inhibitor 1	CDKN1A	5.00
Cerebral dopamine neurotrophic factor	CDNF	1.15
Cell adhesion molecule-related/down-regulated by oncogenes	CDON	12.05
Corneodesmosin	CDSN	88.62
Carcinoembryonic antigen-related cell adhesion molecule 1	CEACAM1	5.80
Carcinoembryonic antigen-related cell adhesion molecule 3	CEACAM3	0.00
Carcinoembryonic antigen	CEA	9.06
Carcinoembryonic antigenrelated cell adhesion molecule 8	CEACAM8	24.08
Liver carboxylesterase 1	CES1	23.17
Cocaine esterase	CES2	51.63
Centrin-2	CETN2	8.57
Cryptic protein	CFC1	6.09
Complement factor H-related protein 5	CFHR5	36.31
Glycoprotein hormones alpha chain	CGA	11.91
Chitinase-3-like protein 1	CHI3L1	8.81
Chitotriosidase-1	CHIT1	29.03
Neural cell adhesion molecule L1-like protein	CHL1	33.86
Chordin-like protein 2	CHRDL2	7.07
Cytoskeleton-associated protein 4	CKAP4	17.79
C-type lectin domain family 10 member A	CLEC10A	7.99
C-type lectin domain family 11 member A	CLEC11A	9.88
C-type lectin domain family 14 member A	CLEC14A	11.47
C-type lectin domain family 1 member A	CLEC1A	14.40
C-type lectin domain family 1 member B	CLEC1B	15.70
C-type lectin domain family 4 member A	CLEC4A	6.42
C-type lectin domain family 4 member C	CLEC4C	4.53
C-type lectin domain family 4 member D	CLEC4D	6.58
C-type lectin domain family 4 member G	CLEC4G	6.12
C-type lectin domain family 5 member A	CLEC5A	36.86
C-type lectin domain family 6 member A	CLEC6A	9.26
C-type lectin domain family 7 member A	CLEC7A	10.23
CMRF35-like molecule 1	CLM-1	11.57
CMRF35-like molecule 6	CLM-6	5.53
CXADR-like membrane protein	CLMP	10.84
Claspin	CLSPN	0.00
Calsyntenin-1	CLSTN1	10.80
Calsyntenin-2	CLSTN2	5.12
Calsyntenin-3	CLSTN3	15.46

Clusterin-like protein 1	CLUL1	6.34
Beta-Ala-His dipeptidase	CNDP1	31.57
Contactin-1	CNTN1	8.70
Contactin-2	CNTN2	19.63
Contactin-4	CNTN4	4.47
Contactin-5	CNTN5	9.31
Contactin-associated protein-like 2	CNTNAP2	4.45
Cochlin	COCH	14.96
Collagen alpha-1(XVIII) chain	COL18A1	33.42
Collagen alpha-1(I) chain	COL1A1	10.09
Collagen alpha-1(IV) chain	COL4A1	21.59
Collagen type IV alpha-3-binding protein	COL4A3BP	14.22
Collectin-12	COLEC12	10.48
Cartilage oligomeric matrix protein	COMP	43.95
Catechol O-methyltransferase	COMT	22.81
Carboxypeptidase A1	CPA1	7.74
Carboxypeptidase A2	CPA2	9.17
Carboxypeptidase B	CPB1	7.25
Carboxypeptidase E	CPE	13.11
Carboxypeptidase M	CPM	5.43
Probable carboxypeptidase X1	CPXM1	19.28
Complement receptor type 2	CR2	50.24
Death domain-containing protein	CRADD	17.91
Cysteine-rich with EGF-like domain protein 2	CRELD2	9.62
Corticoliberin	CRH	18.39
Corticotropin-releasing factor-binding protein	CRHBP	6.89
Cysteine-rich motor neuron 1 protein	CRIM1	12.35
Cysteine-rich protein 2	CRIP2	14.35
Cysteine-rich secretory protein 2	CRISP2	20.30
Crk-like protein	CRKL	34.34
Cornulin	CRNN	7.60
Cartilage acidic protein 1	CRTAC1	39.68
Cytotoxic and regulatory T-cell molecule	CRTAM	11.67
Cone-rod homeobox protein	CRX	19.26
Macrophage colony-stimulating factor 1	CSF-1	5.95
Casein kinase I isoform delta	CSNK1D	11.88
Cystatin-C	CST3	35.52
Cystatin D	CST5	6.60
Cystatin-M	CST6	10.31
Cystatin-B	CSTB	7.93
Cardiotrophin-1	CTF1	0.00

Chymotrypsin C	CTRC	8.73
Dipeptidyl peptidase 1	CTSC	17.96
Cathepsin D	CTSD	7.38
Cathepsin F	CTSF	12.50
Pro-cathepsin H	CTSH	5.73
Cathepsin L1	CTSL1	1.85
Cathepsin O	CTSO	4.57
Cathepsin S	CTSS	2.69
Cathepsin L2	CTSV	7.58
Cathepsin Z	CTSZ	8.36
Fractalkine	CX3CL1	5.30
Coxsackievirus and adenovirus receptor	CXADR	7.06
C-X-C motif chemokine 1	CXCL1	6.63
C-X-C motif chemokine 10	CXCL10	15.78
C-X-C motif chemokine 11	CXCL11	17.64
Stromal cell-derived factor 1	CXCL12	10.12
C-X-C motif chemokine 13	CXCL13	6.75
C-X-C motif chemokine 16	CXCL16	8.17
C-X-C motif chemokine 5	CXCL5	11.31
C-X-C motif chemokine 6	CXCL6	11.71
C-X-C motif chemokine 9	CXCL9	12.39
VEGF-co regulated chemokine 1	CXL17	14.21
Protein CYR61	CYR61	10.86
Disabled homolog 2	DAB2	56.73
Dystroglycan	DAG1	10.68
Dual adapter for phosphotyrosine and 3-phosphotyrosine and 3-phosphoinositide	DAPP1	11.85
Discoidin, CUB and LCCL domain-containing protein 2	DCBLD2	6.97
Decorin	DCN	6.29
Dynactin subunit 1	DCTN1	13.73
Dynactin subunit 2	DCTN2	25.33
N(G),N(G)-dimethylarginine dimethylaminohydrolase 1	DDAH1	9.01
Aromatic-L-amino-acid decarboxylase	DDC	3.73
Epithelial discoidin domain-containing receptor 1	DDR1	6.99
Probable ATP-dependent RNA helicase DDX58	DDX58	9.94
2,4-dienoyl-CoA reductase, mitochondrial	DECR1	84.85
Neutrophil defensin 1	DEFA1	3.63
Beta-defensin 4A	DEFB4A	44.75
DNA fragmentation factor subunit alpha	DFFA	6.17
Diacylglycerol kinase zeta	DGKZ	0.00
Diablo homolog, mitochondrial	DIABLO	35.44

Dickkopf-related protein 1	Dkk-1	9.75
Dickkopf-related protein 3	DKK3	12.34
Dickkopf-related protein 4	Dkk-4	8.19
Dickkopf-like protein 1	DKKL1	8.52
Protein delta homolog 1	DLK-1	10.66
Delta-like protein 1	DLL1	8.34
DnaJ homolog subfamily B member 1	DNAJB1	22.30
Delta and Notch-like epidermal growth factor-related receptor	DNER	5.97
Dipeptidase 1	DPEP1	19.31
Dipeptidase 2	DPEP2	12.15
Inactive dipeptidyl peptidase 10	DPP10	3.24
Dipeptidyl peptidase 4	DPP4	36.47
Dipeptidyl aminopeptidase-like protein 6	DPP6	12.47
Dipeptidyl peptidase 2	DPP7	14.14
Draxin	DRAXIN	10.22
Desmocollin-2	DSC2	11.89
Desmoglein-3	DSG3	13.83
Desmoglein-4	DSG4	20.98
Dual specificity protein phosphatase 3	DUSP3	11.08
Endothelin-converting enzyme 1	ECE1	16.91
Tumor necrosis factor receptor superfamily member 27	EDA2R	12.12
Tumor necrosis factor receptor superfamily member EDAR	EDAR	9.15
EGF-like repeat and discoidin I-like domain-containing protein 3	EDIL3	7.92
EGF-containing fibulin-like extracellular matrix protein 1	EFEMP1	43.56
Ephrin-A4	EFNA4	9.18
Pro-epidermal growth factor	EGF	5.83
Epidermal growth factor-like protein 7	EGFL7	23.12
Epidermal growth factor receptor	EGFR	9.10
Egl nine homolog 1	EGLN1	10.67
Eukaryotic translation initiation factor 4B	EIF4B	15.17
Eukaryotic translation initiation factor 4 gamma 1	EIF4G1	10.35
Eukaryotic translation initiation factor 5A-1	EIF5A	0.00
Protein enabled homolog	ENAH	18.64
Endoglin	ENG	29.37
Gamma-enolase	ENO2	12.95
Ectonucleotide pyrophosphatase/phosphodiesterase family member 2 or Autotaxin	ENPP2	9.61
Ectonucleotide pyrophosphatase/phosphodiesterase family member 7	ENPP7	8.42
Protein S100-A12	EN-RAGE	4.50
Ectonucleoside triphosphate diphosphohydrolase 2	ENTPD2	14.58
Ectonucleoside triphosphate diphosphohydrolase 5	ENTPD5	11.68

Ectonucleoside triphosphate diphosphohydrolase 6	ENTPD6	13.43
Epithelial cell adhesion molecule	Ep-CAM	12.71
Ephrin type-A receptor 10	EPHA10	0.00
Ephrin type-A receptor 2	EPHA2	9.43
Ephrin type-B receptor 4	EPHB4	8.82
Ephrin type-B receptor 6	EPHB6	8.87
Erythropoietin	EPO	5.25
Receptor tyrosine-protein kinase erbB-2	ErbB2/HER2	7.15
Erbin	ERBIN	21.71
Receptor tyrosine-protein kinase erbB-3	ErbB3/HER3	9.50
Receptor tyrosine-protein kinase erbB-4	ErbB4/HER4	7.78
Proepiregulin	EREG	6.85
Endothelial cell-selective adhesion molecule	ESAM	9.30
Endothelial cell-specific molecule 1	ESM-1	12.30
Ezrin	EZR	12.17
Coagulation factor XI	F11	36.78
Coagulation factor VII	F7	33.74
Fatty acid-binding protein, intestinal	FABP2	10.89
Fatty acid-binding protein, adipocyte	FABP4	11.38
Fatty acid-binding protein 9	FABP9	13.99
FAS-associated death domain protein	FADD	7.21
Protein FAM19A5	FAM19A5	21.52
Protein FAM3B	FAM3B	12.53
Protein FAM3C	FAM3C	11.36
Prolyl endopeptidase FAP	FAP	36.57
Tumor necrosis factor receptor superfamily member 6	FAS	10.47
Fas antigen ligand	FasL	4.40
Nuclear factor of activated T-cells, cytoplasmic 1	NFATC1	19.12
Fructose-1,6-bisphosphatase 1	FBP1	28.77
Immunoglobulin alpha Fc receptor	FCAR	8.28
Low affinity immunoglobulin epsilon Fc receptor	FCER2	17.61
Low affinity immunoglobulin gamma Fc region receptor II-a	FCGR2A	36.86
Low affinity immunoglobulin gamma Fc region receptor III-B	FCGR3B	39.98
Ficolin-2	FCN2	40.94
Fc receptor-like protein 1	FCRL1	5.41
Fc receptor-like protein 2	FcRL2	7.63
Fc receptor-like protein 3	FCRL3	1.39
Fc receptor-like protein 5	FCRL5	8.49
Fc receptor-like protein 6	FCRL6	4.58
Fc receptor-like B	FCRLB	21.62
Tyrosine-protein kinase Fes/Fps	FES	27.86

Fetuin-B	FETUB	36.90
Fibroblast growth factor 19	FGF-19	13.31
Fibroblast growth factor 2	FGF2	9.63
Fibroblast growth factor 21	FGF-21	18.52
Fibroblast growth factor 23	FGF-23	9.23
Fibroblast growth factor 5	FGF-5	0.00
Fibroblast growth factor-binding protein 1	FGF-BP1	5.88
Fibroblast growth factor receptor 2	FGFR2	10.51
Tyrosine-protein kinase Fgr	FGR	20.24
Bis(5'-adenosyl)-triphosphatase	FHIT	14.36
Peptidyl-prolyl cis-trans isomerase FKBP1B	FKBP1B	27.92
Peptidyl-prolyl cis-trans isomerase FKBP4	FKBP4	19.04
Peptidyl-prolyl cis-trans isomerase FKBP5	FKBP5	15.40
Peptidyl-prolyl cis-trans isomerase FKBP7	FKBP7	7.36
Friend leukemia integration 1 transcription factor	FLI1	32.47
Leucine-rich repeat transmembrane protein FLRT2	FLRT2	10.20
Fms-related tyrosine kinase 3 ligand	Flt3L	10.31
Folate receptor beta	FOLR2	10.48
Protein fosB	FOSB	28.00
Forkhead box protein O1	FOXO1	12.90
Folate receptor alpha	FR-alpha	9.37
Folate receptor gamma	FR-gamma	6.47
Follistatin	FS	13.82
Follistatin-related protein 3	FSTL3	10.81
Tissue alpha-L-fucosidase	FUCA1	12.79
Furin	FUR	6.09
Galactoside 3(4)-L-fucosyltransferase,Alpha-(1,3)-fucosyltransferase 3/5	FUT3/5	12.83
Alpha-(1,6)-fucosyltransferase	FUT8	14.26
FXYD domain-containing ion transport regulator 5	FXYD5	9.34
Galanin peptides	GAL	10.20
Galectin-1	Gal-1	6.90
Galectin-3	Gal-3	9.31
Galectin-4	Gal-4	9.74
Galectin-8	gal-8	10.17
Galectin-9	Gal-9	7.60
Polypeptide N-acetylgalactosaminyltransferase 10	GALNT10	24.46
Polypeptide N-acetylgalactosaminyltransferase 2	GALNT2	12.39
Polypeptide N-acetylgalactosaminyltransferase 3	GALNT3	8.47
Growth arrest-specific protein 6	GAS6	43.04
Guanylate-binding protein 2	GBP2	0.04
Glucagon	GCG	34.48

Beta-1,3-galactosyl-O-glycosyl-glycoprotein beta-1,6-N-acetylglucosaminyltransferase	GCNT1	30.95
Glypican-5	GCP5	6.85
Granulocyte Colony-Stimulating Factor	G-CSF	8.59
Growth/differentiation factor 15	GDF-15	11.99
Growth/differentiation factor 2	GDF-2	4.74
Growth/differentiation factor 8	GDF-8	9.10
Glial cell line-derived neurotrophic factor	GDNF	0.00
GDNF family receptor alpha-3	GDNFR-alpha-3	8.39
GDNF family receptor alpha-2	GFRA2	8.05
GDNF family receptor alpha-1	GFR-alpha-1	11.48
Glutathione hydrolase 5 proenzyme	GGT5	14.08
Growth hormone	GH	35.07
Growth hormone variant	GH2	52.49
Appetite-regulating hormone	GHRL	10.80
Gastric intrinsic factor	GIF	9.40
Gastrokine	GKN1	6.14
Beta-galactosidase	GLB1	12.55
Lactoylglutathione lyase	GLO1	15.41
Glutaredoxin-1	GLRX	55.20
Granulocyte-macrophage colony-stimulating factor receptor subunit alpha	GM-CSF-R-alpha	10.66
Granulysin	GNLY	16.29
Platelet glycoprotein Ib alpha chain	GP1BA	42.58
Human GPVI Antibody	GPVI	17.44
Glypican-1	GPC1	12.76
Transmembrane glycoprotein NMB	GPNMB	11.89
GRB2-related adapter protein 2	GRAP2	42.93
Granulins	GRN	8.90
Gamma-secretase-activating protein	GSAP	14.12
Glutathione S-transferase P	GSTP1	0.00
Gastrotropin	GT	15.24
Beta-glucuronidase	GUSB	11.73
Granzyme A	GZMA	10.37
Granzyme B	GZMB	13.63
Granzyme H	GZMH	13.37
Hydroxyacylglutathione hydrolase, mitochondrial	HAGH	14.36
Hydroxyacid oxidase 1	HAOX1	18.04
Hepatitis A virus cellular receptor 2	HAVCR2	8.76
Proheparin-binding EGF-like growth factor	HB-EGF	4.17
Hematopoietic lineage cell-specific protein	HCLS1	10.57

Hepatoma-derived growth factor	HDGF	10.83
Protein HEXIM1	HEXIM1	8.60
Hepatocyte growth factor	HGF	12.61
Kallikrein-11	hK11	9.67
Kallikrein-14	hK14	8.53
Kallikrein-8	hK8	5.38
Heme oxygenase 2	HMOX2	45.76
Histamine N-methyltransferase	HNMT	4.15
Heme oxygenase 1	HO-1	9.05
Osteoclast-associated immunoglobulin-like receptor	hOSCAR	3.38
Hematopoietic prostaglandin D synthase	HPGDS	10.06
Heparan sulfate glucosamine 3-O-sulfotransferase 3B1	HS3ST3B1	14.35
Heparan-sulfate 6-O-sulfotransferase 1	HS6ST1	22.67
Corticosteroid 11-beta-dehydrogenase isozyme 1	HSD11B1	7.47
Heat shock 27 kDa protein	HSP 27	6.96
Endoplasmin	HSP90B1	0.00
Serine protease HTRA2, mitochondrial	HTRA2	8.68
Islet cell autoantigen 1	ICA1	12.05
Intercellular adhesion molecule 1	ICAM1	38.36
Intercellular adhesion molecule 2	ICAM-2	10.21
Intercellular adhesion molecule 3	ICAM3	28.83
ICOS ligand	ICOSLG	7.38
Alpha-L-iduronidase	IDUA	7.00
Gamma-interferon-inducible lysosomal thiol reductase	IFI30	9.07
Interferon gamma	IFN-gamma	0.00
Interferon gamma receptor 1	IFN-gamma-R1	7.78
Interferon lambda-1	IFNL1	10.72
Interferon lambda receptor 1	IFNLR1	4.72
Insulin-like growth factor 1 receptor	IGF1R	11.56
Cation-independent mannose-6-phosphate receptor	IGF2R	7.10
Insulin-like growth factor-binding protein 1	IGFBP-1	16.93
Insulin-like growth factor-binding protein 2	IGFBP-2	9.19
Insulin-like growth factor-binding protein 3	IGFBP3	36.56
Insulin-like growth factor-binding protein 6	IGFBP6	37.66
Insulin-like growth factor-binding protein 7	IGFBP-7	12.26
Insulin-like growth factor-binding protein-like 1	IGFBPL1	4.36
Low affinity immunoglobulin gamma Fc region receptor II-b	IgG Fc receptor II-b	8.63
Ig lambda-2 chain C regions	IGLC2	34.05
Immunoglobulin superfamily member 3	IGSF3	14.96
Zinc finger protein Helios	IKZF2	20.51

Interleukin-10	IL10	11.47
Interleukin-10 receptor subunit alpha	IL-10RA	5.08
Interleukin-10 receptor subunit beta	IL-10RB	9.98
Interleukin-12	IL-12	8.38
Interleukin-12 subunit beta	IL-12B	12.00
Interleukin-12 receptor subunit beta-1	IL12RB1	5.02
Interleukin-13	IL-13	0.00
Interleukin-13 receptor subunit alpha-1	IL13RA1	11.10
Interleukin-15	IL15	7.98
Interleukin-15 receptor subunit alpha	IL-15RA	0.00
Pro-interleukin-16	IL16	8.98
Interleukin-17A	IL-17A	6.35
Interleukin-17C	IL-17C	0.00
Interleukin-17D	IL-17D	7.44
Interleukin-17 receptor A	IL-17RA	22.15
Interleukin-17 receptor B	IL17RB	19.05
Interleukin-18	IL-18	4.17
Interleukin-18-binding protein	IL-18BP	9.27
Interleukin-18 receptor 1	IL-18R1	11.26
Interleukin-1 alpha	IL-1 alpha	0.00
Interleukin-1 receptor antagonist protein	IL-1ra	6.55
Interleukin-1 receptor-like 2	IL1RL2	4.53
Interleukin-1 receptor type 1	IL-1RT1	9.09
Interleukin-1 receptor type 2	IL-1RT2	9.17
Interleukin-2	IL-2	0.00
Interleukin-20	IL-20	0.00
Interleukin-20 receptor subunit alpha	IL-20RA	0.00
Interleukin-22 receptor subunit alpha-1	IL-22 RA1	0.00
Interleukin-24	IL-24	0.00
Interleukin-27	IL-27	4.77
Interleukin-2 receptor subunit alpha	IL2-RA	8.99
Interleukin-2 receptor subunit beta	IL-2RB	0.00
Interleukin-32	IL32	9.20
Interleukin-33	IL-33	0.00
Interleukin-3 receptor subunit alpha	IL3RA	6.73
Interleukin-4	IL-4	0.00
Interleukin-4 receptor subunit alpha	IL-4RA	10.64
Interleukin-5	IL5	10.02
Interleukin-5 receptor subunit alpha	IL-5R-alpha	14.06
Interleukin-6	IL6	5.64
Interleukin-6 receptor subunit alpha	IL-6RA	7.13

Interleukin-7	IL-7	7.62
Interleukin-7 receptor subunit alpha	IL7R	43.38
Interleukin-8	IL-8	14.52
Integrin-linked kinase-associated serine/threonine phosphatase 2C	ILKAP	21.08
Inositol monophosphatase 1	IMPA1	9.46
Inhibitor of growth protein 1	ING1	17.09
Inhibin beta C chain	INHBC	13.70
Phosphatidylinositol 3,4,5-trisphosphate 5-phosphatase 2	INPPL1	17.11
Ras GTPase-activating-like protein IQGAP2	IQGAP2	17.81
Interleukin-1 receptor-associated kinase 1	IRAK1	15.91
Interleukin-1 receptor-associated kinase 4	IRAK4	5.85
Interferon regulatory factor 9	IRF9	7.96
Immunoglobulin superfamily containing leucine-rich repeat protein 2	ISLR2	16.35
Integrin alpha-11	ITGA11	10.77
Integrin alpha-5	ITGA5	14.82
Integrin alpha-6	ITGA6	12.76
Integrin alpha-M	ITGAM	4.92
Integrin alpha-V	ITGAV	9.92
Integrin beta-1	ITGB1	7.87
Integrin beta-1-binding protein 1	ITGB1BP1	37.06
Melusin	ITGB1BP2	12.13
Integrin beta-2	ITGB2	93.75
Integrin beta-5	ITGB5	4.31
Integrin beta-6	ITGB6	12.17
Integrin beta-7	ITGB7	1.61
Integral membrane protein 2A	ITM2A	41.01
Junctional adhesion molecule A	JAM-A	107.66
Junctional adhesion molecule B	JAM-B	9.66
Transcription factor AP-1	JUN	0.00
Kazal-type serine protease inhibitor domain-containing protein 1	KAZALD1	15.52
KIF1-binding protein	KIF1BP	16.27
Kidney Injury Molecule	KIM1	3.07
Killer cell immunoglobulin-like receptor 2DL3	KIR2DL3	15.14
Kin of IRRE-like protein 2	KIRREL2	6.10
Mast/stem cell growth factor receptor Kit	KIT	28.91
Beta-klotho	KLB	11.55
Kallikrein-10	KLK10	32.21
Kallikrein-12	KLK12	25.84
Kallikrein-13	KLK13	13.56
Kallikrein-6	KLK6	8.80
Natural killer cells antigen CD94	KLRD1	11.82

Importin subunit alpha-5	KPNA1	0.00
Keratin, type I cytoskeletal 19	KRT19	19.81
Kynurenine--oxoglutarate transaminase 1	KYAT1	10.35
Kynureninase	KYNU	7.65
Lymphocyte activation gene 3 protein	LAG3	8.48
Leukocyte-associated immunoglobulin-like receptor 1	LAIR1	9.65
Leukocyte-associated immunoglobulin-like receptor 2	LAIR-2	15.12
Laminin subunit alpha-4	LAMA4	3.23
Lysosome-associated membrane glycoprotein 3	LAMP3	9.16
Latency-associated peptide transforming growth factor beta-1	LAP TGF-beta-1	11.78
Linker for activation of T-cells family member 1	LAT	8.95
Linker for activation of T-cells family member 2	LAT2	16.48
Layilin	LAYN	13.25
Neutrophil gelatinase-associated lipocalin	LCN2	18.04
Low-density lipoprotein receptor	LDL receptor	14.95
Leptin	LEP	2.93
Leptin receptor	LEPR	13.65
Galectin-7	LGALS7	7.26
Legumain	LGMN	9.87
Lutropin subunit beta	LHB	9.60
Leukemia inhibitory factor	LIF	0.00
Leukemia inhibitory factor receptor	LIF-R	4.44
Leukocyte immunoglobulin-like receptor subfamily A member 5	LILRA5	6.52
Leukocyte immunoglobulin-like receptor subfamily B member 1	LILRB1	36.26
Leukocyte immunoglobulin-like receptor subfamily B member 2	LILRB2	31.54
Leukocyte immunoglobulin-like receptor subfamily B member 4	LILRB4	4.04
Leukocyte immunoglobulin-like receptor subfamily B member 5	LILRB5	33.34
Lectin-like oxidized LDL receptor 1	LOX-1	21.63
Lipoprotein lipase	LPL	3.54
Leucine-rich repeats and immunoglobulin-like domains protein 1	LRIG1	4.82
Lymphoid-restricted membrane protein	LRMP	9.60
Prolow-density lipoprotein receptor-related protein 1	LRP1	17.86
Low-density lipoprotein receptor-related protein 11	LRP11	7.21
Leucine-rich repeat neuronal protein 1	LRRN1	19.54
Leukotriene A-4 hydrolase	LTA4H	29.79
Latent-transforming growth factor beta-binding protein 2	LTBP2	0.94
Lymphotoxin-beta receptor	LTBR	11.76
Latexin	LXN	2.89
Lymphocyte antigen 75	LY75	4.59
T-lymphocyte surface antigen Ly-9	LY9	7.68

Cell growth-regulating nucleolar protein	LYAR	15.52
Tyrosine-protein kinase Lyn	LYN	10.23
Ly6/PLAUR domain-containing protein 1	LYPD1	1.34
Ly6/PLAUR domain-containing protein 3	LYPD3	9.72
Lymphatic vessel endothelial hyaluronic acid receptor 1	LYVE1	29.95
Mitotic spindle assembly checkpoint protein MAD1	MAD1L1	21.51
Mothers against decapentaplegic homolog 5	MAD homolog 5	3.82
Macrophage erythroblast attacher	MAEA	51.99
Melanoma-associated antigen D1	MAGED1	33.61
Mesencephalic astrocyte-derived neurotrophic factor	MANF	3.55
Dual specificity mitogen-activated protein kinase kinase 6	MAP2K6	14.03
Mitogen-activated protein kinase kinase kinase kinase 5	MAP4K5	15.86
Microtubule-associated protein Tau	MAPT	0.00
Macrophage receptor MARCO	MARCO	4.11
Mannan-binding lectin serine protease 1	MASP1	8.54
Matrilin-2	MATN2	10.63
Matrilin-3	MATN3	7.25
Protein max	MAX	36.05
Myoglobin	MB	7.87
Mannose-binding protein C	MBL2	65.70
Multiple coagulation factor deficiency protein 2	MCFD2	10.58
Monocyte chemotactic protein 1	MCP-1	11.38
Monocyte chemotactic protein 2	MCP-2	10.74
Monocyte chemotactic protein 3	MCP-3	9.75
Monocyte chemotactic protein 4	MCP-4	13.79
MAM domain-containing glycosylphosphatidylinositol anchor protein 1	MDGA1	12.34
Multiple epidermal growth factor-like domains protein 9	MEGF9	27.38
Meprin A subunit beta	MEP1B	10.96
Matrix extracellular phosphoglycoprotein	MEPE	7.54
Tyrosine-protein kinase Mer	MERTK	1.96
LDLR chaperone MESD	MESDC2	11.90
Hepatocyte growth factor receptor	MET	29.75
Methionine aminopeptidase 1	METAP1	4.98
Methionine aminopeptidase 1D, mitochondrial	METAP1D	25.72
Methionine aminopeptidase 2	MetAP 2	10.38
Meteorin-like protein	METRNL	4.00
Microfibrillar-associated protein 5	MFAP5	27.58
Lactadherin	MFGE8	13.67
Methylated-DNA--protein-cysteine methyltransferase	MGMT	9.14
Melanoma-derived growth regulatory protein	MIA	7.45

MHC class I polypeptide-related sequence A/B	MIC-A/B	7.41
Macrophage migration inhibitory factor	MIF	20.78
Allergin-1	MILR1	5.35
Midkine	MK	11.44
Matrix metalloproteinase-1	MMP-1	12.13
Matrix metalloproteinase-10	MMP-10	7.35
Matrix metalloproteinase-12	MMP-12	2.67
Matrix metalloproteinase-2	MMP-2	8.93
Matrix metalloproteinase-3	MMP-3	12.66
Matrix metalloproteinase-7	MMP-7	8.05
Matrix metalloproteinase-9	MMP-9	23.37
Myelin-oligodendrocyte glycoprotein	MOG	15.89
Myeloperoxidase	MPO	72.59
C-type mannose receptor 2	MRC2	14.00
Mesothelin	MSLN	10.19
Beta-microseminoprotein	MSMB	8.37
Macrophage scavenger receptor types I and II	MSR1	7.73
Mucin-16	MUC-16	7.83
Mevalonate kinase	MVK	16.81
Myocilin	MYOC	7.94
NKG2D ligand 2	N2DL-2	3.32
N-alpha-acetyltransferase 10	NAA10	23.10
N-acylethanolamine-hydrolyzing acid amidase	NAAA	10.76
NAD kinase	NADK	5.24
Neuroblastoma suppressor of tumorigenicity 1	NBL1	5.75
Nibrin	NBN	22.32
Neural cell adhesion molecule 1	NCAM1	31.83
Neurocan core protein	NCAN	7.66
Neutral ceramidase	N-CDase	6.89
Neutrophil cytosol factor 2	NCF2	28.76
Nicalin	NCLN	0.00
Natural cytotoxicity triggering receptor 1	NCR1	6.21
Protein NDRG1	NDRG1	6.01
Nectin-2	NECTIN2	13.90
Neurofilament light polypeptide	NEFL	17.98
NF-kappa-B essential modulator	NEMO	44.21
Neprilysin	NEP	7.37
Merlin	NF2	9.83
Nuclear factor of activated T-cells, cytoplasmic 3	NFATC3	4.14
NF-kappa-B inhibitor epsilon	NFKBIE	13.48
Nidogen-1	NID1	39.00

Nidogen-2	NID2	6.46
Ninjurin-1	NINJ1	3.17
Nicotinamide/nicotinic acid mononucleotide adenylyltransferase 1	NMNAT1	10.44
Nodal modulator 1	NOMO1	12.44
Nitric oxide synthase, endothelial	NOS3	12.01
Neurogenic locus notch homolog protein 1	NOTCH1	36.58
Neurogenic locus notch homolog protein 3	Notch 3	7.97
Protein NOV homolog	NOV	6.92
Neural proliferation differentiation and control protein 1	NPDC1	11.94
Nucleophosmin	NPM1	13.36
C-type natriuretic peptide	NPPC	16.62
Neuronal pentraxin receptor	NPTXR	5.58
Ribosyldihydronicotinamide dehydrogenase (quinone)	NQO2	15.07
Neuronal cell adhesion molecule	Nr-CAM	4.47
Neuropilin-1	NRP1	25.78
Neuropilin-2	NRP2	14.92
Neurturin	NRTN	4.78
Neurotrophin-3	NT-3	17.92
Neurotrophin-4	NTF4	11.54
N-terminal prohormone brain natriuretic peptide	NT-proBNP	14.23
BDNF/NT-3 growth factors receptor	NTRK2	5.98
NT-3 growth factor receptor	NTRK3	5.95
NEDD8 ultimate buster 1	NUB1	11.67
Nucleobindin-2	NUCB2	22.88
ADP-sugar pyrophosphatase	NUDT5	10.08
Neurexophilin-1	NXPH1	0.00
Osteomodulin	OMD	18.02
Oligodendrocyte-myelin glycoprotein	OMG	19.26
Osteoprotegerin	OPG	8.59
Osteopontin	OPN	14.43
Opticin	OPTC	15.05
Oncostatin-M	OSM	12.37
Oncostatin-M-specific receptor subunit beta	OSMR	23.94
Protein disulfide-isomerase	P4HB	10.83
Protein-arginine deiminase type-2	PADI2	1.66
Glycodelin	PAEP	12.76
Phosphoprotein associated with glycosphingolipid-enriched microdomains 1	PAG1	59.29
Plasminogen activator inhibitor 1	PAI	7.37
Serine/threonine-protein kinase PAK 4	PAK4	12.37
Peptidyl-glycine alpha-amidating monooxygenase	PAM	34.38
Inactive serine protease PAMR1	PAMR1	14.64

Pappalysin-1	PAPPA	8.09
Proteinase-activated receptor 1	PAR-1	3.36
Protein deglycase DJ-1	PARK7	11.00
Poly (ADP-ribose) polymerase 1	PARP-1	19.34
Protocadherin-17	PCDH17	19.09
Procollagen C-endopeptidase enhancer 1	PCOLCE	38.31
Proprotein convertase subtilisin/kexin type 9	PCSK9	9.28
Programmed cell death protein 1	PDCD1	28.13
Platelet-derived growth factor C	PDGFC	19.55
Platelet-derived growth factor receptor alpha	PDGF-R-alpha	8.47
Platelet-derived growth factor receptor beta	PDGFRB	5.63
Platelet-derived growth factor subunit A	PDGF subunit A	13.45
Platelet-derived growth factor subunit B	PDGF subunit B	14.61
Programmed cell death 1 ligand 1	PD-L1	6.44
Programmed cell death 1 ligand 2	PD-L2	3.48
(Pyruvate dehydrogenase (acetyl-transferring))-phosphatase 1, mitochondria	PDP1	0.00
Platelet endothelial aggregation receptor 1	PEAR1	13.20
Phosphatidylethanolamine-binding protein 1	PEBP1	12.56
Platelet endothelial cell adhesion molecule	PECAM-1	85.23
Prefoldin subunit 2	PFDN2	9.69
ATP-dependent 6-phosphofructokinase, muscle type	PFKM	7.31
Placenta growth factor	PGF	5.13
Peptidoglycan recognition protein 1	PGLYRP1	7.77
Phosphoethanolamine/phosphocholine phosphatase	PHOSPHO1	14.44
Elafin	PI3	8.57
Polymeric immunoglobulin receptor	PIgR	4.64
Phosphoinositide 3-kinase adapter protein 1	PIK3AP1	6.41
Paired immunoglobulin-like type 2 receptor alpha	PILRA	14.40
Paired immunoglobulin-like type 2 receptor beta	PILRB	8.11
Group 10 secretory phospholipase A2	PLA2G10	12.72
Cytosolic phospholipase A2	PLA2G4A	10.35
Platelet-activating factor acetylhydrolase	PLA2G7	18.94
Perlecan	PLC	7.92
Perilipin-1	PLIN1	14.83
Phospholipid transfer protein	PLTP	28.67
Plexin domain-containing protein 1	PLXDC1	8.04
Plexin-A4	PLXNA4	19.35
Plexin-B1	PLXNB1	9.18
Plexin-B2	PLXNB2	22.50

Plexin-B3	PLXNB3	11.39
Phosphomevalonate kinase	PMVK	23.92
Podocalyxin	PODXL	12.90
Podocalyxin-like protein 2	PODXL2	17.32
Serum paraoxonase/arylesterase 2	PON2	17.59
Paraoxonase	PON3	11.77
Peptidyl-prolyl cis-trans isomerase B	PPIB	11.82
Protein phosphatase 1B	PPM1B	0.00
Protein phosphatase inhibitor 2	PPP1R2	20.10
Neurabin-2	PPP1R9B	13.46
Calcineurin subunit B type 1	PPP3R1	14.31
Pancreatic prohormone	PPY	8.86
Lysosomal Pro-X carboxypeptidase	PRCP	8.88
Peroxiredoxin-1	PRDX1	6.29
Thioredoxin-dependent peroxide reductase, mitochondrial	PRDX3	14.55
Peroxiredoxin-5, mitochondrial	PRDX5	24.19
Peroxiredoxin-6	PRDX6	21.98
Prolactin regulatory element-binding protein	PREB	6.17
Prolargin	PRELP	3.01
5'-AMP-activated protein kinase subunit beta-1	PRKAB1	19.42
Protein kinase C theta type	PRKCQ	16.71
Interferon-inducible double-stranded RNA-dependent protein kinase activator A	PRKRA	24.14
Vitamin K-dependent protein C	PROC	44.87
Prokineticin-1	PROK1	14.70
Trypsin-2	PRSS2	32.75
Serine protease 27	PRSS27	6.76
Prostasin	PRSS8	1.43
Phosphoribosyltransferase domain-containing protein 1	PRTFDC1	17.94
Protogenin	PRTG	11.79
Myeloblastin	PRTN3	57.28
Pregnancy-specific beta-1-glycoprotein 1	PSG1	14.46
P-selectin glycoprotein ligand 1	PSGL-1	8.43
PC4 and SFRS1-interacting protein	PSIP1	9.04
Proteasome subunit alpha type-1	PSMA1	14.80
Proteasome activator complex subunit 1	PSME1	12.68
Pulmonary surfactant-associated protein D	PSP-D	11.97
Parathyroid hormone/parathyroid hormone-related peptide receptor	PTH1R	11.62
Inactive tyrosine-protein kinase 7	PTK7	32.88
Pleiotrophin	PTN	32.07
Tyrosine-protein phosphatase non-receptor type 1	PTPN1	16.74

Tyrosine-protein phosphatase non-receptor type 6	PTPN6	12.88
Receptor-type tyrosine-protein phosphatase F	PTPRF	10.16
Receptor-type tyrosine-protein phosphatase eta	PTPRJ	18.54
Receptor-type tyrosine-protein phosphatase S	PTPRS	25.30
6-pyruvoyl tetrahydrobiopterin synthase	PTS	16.75
Pentraxin-related protein PTX3	PTX3	15.75
Parvalbumin alpha	PVALB	14.30
Poliovirus receptor	PVR	9.44
Nectin-4	PVRL4	7.54
Paxillin	PXN	18.64
Dihydropteridine reductase	QDPR	10.90
Glutaminyl-peptide cyclotransferase	QPCT	20.65
Receptor for advanced glycosylation end products	RAGE	4.47
Retinoic acid receptor responder protein 1	RARRES1	5.82
Retinoic acid receptor responder protein 2	RARRES2	7.28
Ras GTPase-activating protein 1	RASA1	21.47
Ras association domain-containing protein 2	RASSF2	25.61
Ribokinase	RBKS	16.75
REST corepressor 1	RCOR1	37.53
Lithostathine-1-alpha	REG1A	31.47
Regenerating islet-derived protein 3-alpha	REG3A	0.00
Regenerating islet-derived protein 4	REG4	14.18
Tumor necrosis factor receptor superfamily member 19L	RELT	8.71
Renin	REN	9.62
Proto-oncogene tyrosine-protein kinase receptor Ret	RET	13.90
Resistin	RETN	12.18
Repulsive guidance molecule A	RGMA	7.80
RGM domain family member B	RGMB	10.77
Regulator of G-protein signaling 8	RGS8	0.55
Eosinophil cationic protein	RNASE3	34.59
E3 ubiquitin-protein ligase RNF31	RNF31	7.82
Roundabout homolog 1	ROBO1	7.93
Roundabout homolog 2	ROBO2	12.60
Inactive tyrosine-protein kinase transmembrane receptor ROR1	ROR1	6.40
Ribosomal protein S6 kinase beta-1	RPS6KB1	20.59
Ribonucleoside-diphosphate reductase subunit M2 B	RRM2B	7.89
R-spondin-1	RSPO1	11.85
R-spondin-3	RSPO3	13.54
Reticulon-4 receptor	RTN4R	2.94
Protein S100-A11	S100A11	17.47
Protein S100-A4	S100A4	10.62

Protein S100-P	S100P	8.62
Serum amyloid A-4 protein	SAA4	40.51
Secretory carrier-associated membrane protein 3	SCAMP3	19.76
Scavenger receptor class A member 5	SCARA5	7.86
Lysosome membrane protein 2	SCARB2	7.94
Scavenger receptor class F member 1	SCARF1	10.08
Scavenger receptor class F member 2	SCARF2	14.88
Stem cell factor	SCF	2.78
Secretoglobin family 1A member 1	SCGB1A1	3.98
Secretoglobin family 3A member 1	SCGB3A1	8.83
Secretoglobin family 3A member 2	SCGB3A2	14.54
Syndecan-4	SDC4	11.57
E-selectin	SELE	8.06
L-selectin	SELL	38.90
P-selectin	SELP	51.67
Semaphorin-3F	SEMA3F	9.35
Semaphorin-4C	SEMA4C	7.23
Semaphorin-7A	SEMA7A	9.06
Serpin A12	SERPINA12	6.04
Plasma serine protease inhibitor	SERPINA5	39.20
Thyroxine-binding globulin	SERPINA7	36.48
Serpin A9	SERPINA9	12.03
Serpin B6	SERPINB6	18.40
Serpin B8	SERPINB8	29.57
Seizure 6-like protein	SEZ6L	9.69
Seizure 6-like protein 2	SEZ6L2	13.12
Secreted frizzled-related protein 1	SFRP1	18.57
Secreted frizzled-related protein 3	sFRP-3	14.14
SH2B adapter protein 3	SH2B3	11.79
SH2 domain-containing protein 1A	SH2D1A	7.43
Tyrosine-protein phosphatase non-receptor type substrate 1	SHPS-1	10.27
Sialoadhesin	SIGLEC1	7.90
Sialic acid-binding Ig-like lectin 10	SIGLEC10	5.00
Sialic acid-binding Ig-like lectin 6	SIGLEC6	15.58
Sialic acid-binding Ig-like lectin 7	SIGLEC7	8.29
Sialic acid-binding Ig-like lectin 9	Siglec-9	7.51
Signal-regulatory protein beta-1	SIRPB1	8.59
SIR2-like protein 2	SIRT2	14.60
NAD-dependent protein deacylase sirtuin-5, mitochondrial	SIRT5	22.99
Signaling threshold-regulating transmembrane adapter 1	SIT1	6.42
Src kinase-associated phosphoprotein 1	SKAP1	17.63

Serine/threonine-protein kinase receptor R3	SKR3	7.28
Signaling lymphocytic activation molecule	SLAMF1	0.00
SLAM family member 7	SLAMF7	3.39
SLAM family member 8	SLAMF8	10.78
SLIT and NTRK-like protein 2	SLITRK2	19.45
SLIT and NTRK-like protein 6	SLITRK6	0.00
Mothers against decapentaplegic homolog 1	SMAD1	15.69
SPARC-related modular calcium-binding protein 1	SMOC1	18.10
SPARC-related modular calcium-binding protein 2	SMOC2	14.45
Sphingomyelin phosphodiesterase	SMPD1	8.11
Synaptosomal-associated protein 23	SNAP23	80.75
Synaptosomal-associated protein 29	SNAP29	13.53
Gamma-synuclein	SNCG	26.21
Superoxide dismutase (Cu-Zn)	SOD1	11.26
Superoxide dismutase (Mn), mitochondrial (SOD2)	SOD2	24.25
VPS10 domain-containing receptor SorCS2	SORCS2	18.11
Sortilin	SORT1	8.39
Sclerostin	SOST	11.32
SPARC	SPARC	15.25
SPARC-like protein 1	SPARCL1	26.88
Serine protease inhibitor Kazal-type 1	SPINK1	6.35
Serine protease inhibitor Kazal-type 5	SPINK5	7.38
Kunitz-type protease inhibitor 1	SPINT1	11.87
Kunitz-type protease inhibitor 2	SPINT2	2.99
Testican-1	SPOCK1	10.15
Spondin-1	SPON1	17.65
Spondin-2	SPON2	3.19
Protein sprouty homolog 2	SPRY2	16.40
Proto-oncogene tyrosine-protein kinase Src	SRC	9.80
Signal recognition particle 14 kDa protein	SRP14	16.11
SRSF protein kinase 2	SRPK2	9.83
Scavenger receptor cysteine-rich domain-containing group B protein	SSC4D	6.90
Sulfotransferase 1A1	ST1A1	5.53
ST2 protein	ST2	9.61
CMP-N-acetylneuraminate-beta-galactosamide-alpha-2,3-sialyltransferase 1	ST3GAL1	21.12
Beta-galactoside alpha-2,6-sialyltransferase 1	ST6GAL1	36.38
STAM-binding protein	STAMPB	7.68
Stanniocalcin-1	STC1	6.57
Stress-induced-phosphoprotein 1	STIP1	10.86
Serine/threonine-protein kinase 4	STK4	9.73
Syntaxin-16	STX16	15.96

Syntaxin-6	STX6	12.35
Syntaxin-8	STX8	10.71
Syntaxin-binding protein 3	STXBP3	21.76
Bile salt sulfotransferase	SULT2A1	16.51
Sulfatase-modifying factor 2	SUMF2	8.30
Syndecan-1	SYND1	8.59
Transforming acidic coiled-coil-containing protein 3	TACC3	25.20
Tumor-associated calcium signal transducer 2	TACSTD2	12.84
TRAF family member-associated NF-kappa-B activator	TANK	19.11
Tubulin-folding cofactor B	TBCB	21.55
T-cell leukemia / lymphoma protein 1A	TCL1A	12.08
T-cell leukemia/lymphoma protein 1B	TCL1B	20.72
Transcobalamin-2	TCN2	24.83
Teratocarcinoma-derived growth factor 1	TDGF1	1.96
Tudor and KH domain-containing protein	TDRKH	11.59
Tissue factor	TF	2.27
Trefoil factor 2	TFF2	6.68
Trefoil factor 3	TFF3	8.63
Tissue factor pathway inhibitor	TFPI	7.85
Tissue factor pathway inhibitor 2	TFPI-2	10.20
Transforming growth factor alpha	TGF-alpha	12.53
Transforming growth factor-beta-induced protein ig-h3	TGFBI	38.07
Transforming growth factor beta receptor type 3	TGFBR3	33.44
TGF-beta receptor type-2	TGFR-2	6.30
Protein-glutamine gamma-glutamyltransferase 2	TGM2	10.54
Thrombospondin-2	THBS2	6.68
Thrombospondin-4	THBS4	40.18
Thimet oligopeptidase	THOP1	8.11
Thrombopoietin	THPO	4.04
Thy-1 membrane glycoprotein	THY 1	4.86
Tyrosine-protein kinase receptor Tie-1	TIE1	30.50
Angiopoietin-1 receptor	TIE2	3.24
Fructose-2,6-bisphosphatase TIGAR	TIGAR	27.35
T-cell immunoglobulin and mucin domain-containing protein 4	TIMD4	26.14
Metalloproteinase inhibitor 1	TIMP1	34.91
Metalloproteinase inhibitor 4	TIMP4	10.52
Tubulointerstitial nephritis antigen-like	TINAGL1	7.49
Toll-like receptor 3	TLR3	8.29
Trem-like transcript 2 protein	TLT-2	16.81
Thrombomodulin TM	TM	4.41
Enteropeptidase	TMPRSS15	5.18

Transmembrane protease serine 5	TMPRSS5	8.83
Thymosin beta-10	TMSB10	12.51
Tenascin	TNC	35.55
Tumor necrosis factor	TNF	0.00
TNF-beta	TNFB	9.09
Tumor necrosis factor receptor 1	TNF-R1	5.89
Tumor necrosis factor receptor 2	TNF-R2	7.69
Tumor necrosis factor receptor superfamily member 10A	TNFRSF10A	5.86
Tumor necrosis factor receptor superfamily member 10C	TNFRSF10C	12.93
Tumor necrosis factor receptor superfamily member 11A	TNFRSF11A	3.26
Tumor necrosis factor receptor superfamily member 12A	TNFRSF12A	9.07
Tumor necrosis factor receptor superfamily member 13B	TNFRSF13B	5.98
Tumor necrosis factor receptor superfamily member 13C	TNFRSF13C	0.00
Tumor necrosis factor receptor superfamily member 14	TNFRSF14	16.32
Tumor necrosis factor receptor superfamily member 19	TNFRSF19	10.28
Tumor necrosis factor receptor superfamily member 21	TNFRSF21	8.81
Tumor necrosis factor receptor superfamily member 4	TNFRSF4	6.95
Tumor necrosis factor receptor superfamily member 6B	TNFRSF6B	9.79
Tumor necrosis factor receptor superfamily member 9	TNFRSF9	7.83
Tumor necrosis factor ligand superfamily member 13	TNFSF13	9.97
Tumor necrosis factor ligand superfamily member 13B	TNFSF13B	6.86
Tumor necrosis factor ligand superfamily member 14	TNFSF14	15.82
Troponin I, cardiac muscle	TNNI3	29.73
Tenascin-R	TN-R	5.93
Tenascin-X	TNXB	21.68
DNA topoisomerase 2-beta	TOP2B	28.29
Cellular tumor antigen p53	TP53	6.29
Tissue-type plasminogen activator	t-PA	10.56
Tripeptidyl-peptidase 1	TPP1	11.94
Tubulin polymerization-promoting protein family member 3	TPPP3	8.64
Tryptase alpha/beta-1	TPSAB1	9.85
Transferrin receptor protein 1	TR	14.83
TNF receptor-associated factor 2	TRAF2	5.35
TNF-related apoptosis-inducing ligand	TRAIL	12.60
TNF-related apoptosis-inducing ligand receptor 2	TRAIL-R2	3.37
TNF-related activation-induced cytokine	TRANCE	12.34
Tartrate-resistant acid phosphatase type 5	TR-AP	7.22
Triggering receptor expressed on myeloid cells 1	TREM1	11.35
E3 ubiquitin-protein ligase TRIM21	TRIM21	19.55
Tripartite motif-containing protein 5	TRIM5	3.01
Thyrotropin subunit beta	TSHB	8.81

Thymic stromal lymphopoietin	TSLP	0.00
Tumor necrosis factor (Ligand) superfamily, member 12	TWEAK	11.67
Alpha-taxilin	TXLNA	6.73
Thioredoxin domain-containing protein 5	TXNDC5	24.95
Thymidine phosphorylase	TYMP	15.62
Tyrosine-protein kinase receptor TYRO3	TYRO3	6.55
NEDD8-conjugating enzyme UBE2F	UBE2F	3.48
Uromodulin	UMOD	23.93
Netrin receptor UNC5C	UNC5C	15.38
Urokinase-type plasminogen activator	uPA	28.12
Urokinase plasminogen activator surface receptor	U-PAR	28.33
Ubiquitin carboxyl-terminal hydrolase 8	USP8	19.55
Vesicle-associated membrane protein 5	VAMP5	8.88
Vasohibin-1	VASH1	8.26
Vasorin	VASN	28.35
Vascular cell adhesion protein 1	VCAM1	38.52
Versican core protein	VCAN	9.59
Vascular endothelial growth factor A	VEGF-A	10.57
Vascular endothelial growth factor C	VEGFC	22.76
Vascular endothelial growth factor D	VEGFD	5.87
Vascular endothelial growth factor receptor 2	VEGFR-2	6.67
Vascular endothelial growth factor receptor 3	VEGFR-3	7.26
Vimentin	VIM	7.93
V-set and immunoglobulin domain-containing protein 2	VSIG2	13.20
V-set and immunoglobulin domain-containing protein 4	VSIG4	10.12
V-set and transmembrane domain-containing protein 1	VSTM1	18.21
Brorin	VWC2	14.96
von Willebrand factor	vWF	15.22
Wiskott-Aldrich syndrome protein	WAS	22.89
Wiskott-Aldrich syndrome protein family member 1	WASF1	10.74
Wiskott-Aldrich syndrome protein family member 3	WASF3	28.85
WAP four-disulfide core domain protein 2	WFDC2	6.66
WAP, Kazal, immunoglobulin, Kunitz and NTR domain-containing protein 1	WFIKKN1	6.75
WAP, Kazal, immunoglobulin, Kunitz and NTR domain-containing protein 2	WFIKKN2	12.29
Wnt inhibitory factor 1	WIF-1	11.40
WNT1-inducible-signaling pathway protein 1	WISP-1	9.65
Protein Wnt-9a	WNT9A	9.89
NEDD4-like E3 ubiquitin-protein ligase	WWP2	14.96
Eukaryotic translation initiation factor 4E-binding protein 1	4E-BP1	12.16
5'-nucleotidase	5'-NT	6.33
Lymphotactin	XCL1	5.34

Glycoprotein Xg	XG	13.04
Xaa-Pro aminopeptidase 2	XPNPEP2	12.41
Tyrosine-protein kinase Yes	YES1	23.83
Zinc finger and BTB domain-containing protein 16	ZBTB16	8.54
Zinc finger and BTB domain-containing protein 17	ZBTB17	17.57

Abbreviation: CV, coefficient of variation.
The proteins with more than 30% coefficient variation are marked in grey.

Table S2. The inter-assay coefficient of variation for the 27 proteins tested in the validation set.

Proteins	Gene	CV
Disintegrin and metalloproteinase domain-containing protein 22	ADAM22	9.74
Anterior gradient protein 3	AGR3	9.17
Beta-nerve growth factor	Beta-NGF	13.05
Soluble calcium-activated nucleotidase 1	CANT1	13.34
Caspase-8	CASP-8	4.72
B-cell antigen receptor complex-associated protein beta chain	CD79B	9.72
Cadherin-17	CDH17	15.58
CMRF35-like molecule 1	CLM-1	19.67
Cytotoxic and regulatory T-cell molecule	CRTAM	10.45
Dipeptidyl peptidase 1	CTSC	4.87
Epithelial discoidin domain-containing receptor 1	DDR1	10.69
Ephrin-A4	EFNA4	11.31
Ephrin type-B receptor 6	EPHB6	9.51
Fatty acid-binding protein 9	FABP9	15.99
Leucine-rich repeat transmembrane protein FLRT2	FLRT2	17.13
Heat shock 27 kDa protein	HSP-27	17.17
Endoplasmin	HSP90B1	24.22
Interleukin-6 receptor subunit alpha	IL-6RA	11.62
Leukotriene A-4 hydrolase	LTA4H	17.09
Matrilin-3	MATN3	10.90
Natural cytotoxicity triggering receptor 1	NCR1	11.85
SLAM family member 8	SLAMF8	16.91
Serine protease inhibitor Kazal-type 5	SPINK5	6.91
Transferrin receptor protein 1	TR	11.61
TNF-related activation-induced cytokine	TRANCE	12.15
Netrin receptor UNC5C[#]	UNC5C	-
Wiskott-Aldrich syndrome protein	WAS	14.05

Abbreviation: CV, coefficient of variation.

[#] UNC5C was not included in the Olink Explore 1536 assay in validation set.

Table S3. The comparison of host characteristics between the individuals involved in the discovery and validation set.

Characteristics	Discovery, No. (%) (n=196)	Validation, No. (%) (n=120)	*P*
Age at blood draw, mean (SD), y	59.1 (8.8)	60.1 (8.7)	0.316
Age at diagnosis, mean (SD), y	64.8 (8.5)	65.6 (8.8)	0.579
Age interval (diagnose-blood draw), mean (SD), y	5.6 (2.4)	5.5 (2.5)	0.832
BMI, mean (SD), kg/m^2	24.4 (2.9)	25.2 (4.0)	0.247
WHR, mean (SD),	0.8 (0.1)	0.8 (0.1)	0.631
Family income, %			
<10,000RMB	42 (21.4)	36 (30.0)	0.046
10,000RMB-	82 (41.8)	43 (35.8)	
20,000RMB-	41 (20.9)	32 (26.7)	
≥30,000RMB	31 (15.8)	9 (7.5)	
Educational attainment, %			
≤Elementary school	77 (39.3)	52 (43.3)	0.456
Middle school	53 (27.0)	37 (30.8)	
High school	39 (19.9)	16 (13.3)	
≥College	27 (13.8)	15 (12.5)	
Ever smokers, %	4 (2.0)	8 (6.7)	0.074
Ever drinkers, %	1 (0.5)	3 (2.5)	0.309
Physical activity, MET-hrs/day/yrs	0.9 (1.5)	0.9 (1.5)	0.957
Family history of adenomatous polyposis of colorectum, %	1 (0.5)	0 (0.0)	1.000
Family history of colorectal cancer, %	4 (2.0)	2 (1.7)	1.000
Current aspirin use, %	7 (3.6)	5 (4.2)	1.000
Current peptic ulcer medication use, %	7 (3.6)	3 (2.5)	0.844
Ulcerative colitis, %	1 (0.5)	1 (0.8)	1.000
Diabetes, %	18 (9.2)	9 (7.5)	0.755
Colorectal polyp, %	3 (1.5)	2 (1.7)	1.000
Calcium supply, %	37 (18.9)	37 (30.8)	0.022
Total energy, mean (SD), Kcal	1675.1 (411.6)	1628.2 (398.0)	0.316
Red meat, mean (SD), g/day/1000Kcal	29.8 (20.5)	25.7 (17.2)	0.055
Fat, mean (SD), g/day/1000Kcal	17.7 (6.1)	15.8 (5.5)	0.004

Abbreviation: BMI, body mass index; WHR, waist-to-hip ratio.

Table S4. The associations between CRC risk and selected dichotomized protein markers.

	Discovery				Validation			
**Proteins**	**controls**	**cases**	**OR (95% CI)** *	***P***	**controls**	**cases**	**OR (95%CI)** *	***P***
HSP90B1ǂ	80	68	Ref		30	32	Ref	
	18	30	2.57 (1.19-5.57)	0.017	30	28	0.90 (0.37-2.20)	0.819
LTA4Hǂ	74	52	Ref		30	11	Ref	
	24	46	2.55 (1.30-5.02)	0.006	30	49	7.40 (2.11-25.93)	1.76×10^{-3}

*OR was obtained from conditional logistic regression adjusted for age, educational level, and body mass index.

ǂ The dichotomous cutoffs of HSP90B1 were 0.74428 and -0.89045 in discovery and validation phase, respectively.

ǂ The dichotomous cutoffs of LTA4H were 0.71740 and 1.46485 in discovery and validation phase, respectively.
